# Multivalent Bi‐Specific Nanoerythrosomes: A Two‐Birds‐One‐Stone Approach to Combine Homologous and Active Targeting for Effective Thrombolysis

**DOI:** 10.1002/smll.74578

**Published:** 2026-07-13

**Authors:** Yu Huang, Yilin Yang, Nicole Henry, Boram Gu, Jiahua Wang, Yuanyuan Guo, Colin Longstaff, Simon A. Thom, Yuehua Li, Xiao Yun Xu, Rongjun Chen

**Affiliations:** ^1^ Department of Chemical Engineering, Imperial College London South Kensington Campus London UK; ^2^ Department of Radiology Shanghai Jiao Tong University School of Medicine Affiliated Shanghai Sixth People's Hospital Shanghai P. R. China; ^3^ School of Chemical Engineering Chonnam National University Gwangju Republic of Korea; ^4^ Biotherapeutics Section National Institute for Biological Standards and Control South Mimms Hertfordshire UK; ^5^ National Heart and Lung Institute Imperial College London London UK

**Keywords:** active targeting, computational simulation, effective thrombolysis, homologous targeting, nanoerythrosome

## Abstract

Combining homologous targeting and active targeting to a thrombus is highly desirable in targeted thrombolytic therapy. Here, we report a multivalent bi‐specific nanoerythrosome for thrombus‐targeted delivery of tissue plasminogen activator (tPA) (denoted as mBiNE@tPA) to achieve effective thrombolysis. This bio‐inspired delivery system was fabricated to exploit two main thrombus components by utilizing the natural erythrocyte membrane for homologous targeting to a thrombus and fibrinogen‐derived cyclic arginine‐glycine‐aspartic acid (cRGD) motifs for active targeting to activated platelets. mBiNE@tPA maintained tPA's activity during extended circulation. Its preferential thrombus accumulation, enhanced thrombus penetration, and triggered tPA release locally through the combined homologous and active thrombus targeting were demonstrated. Efficient and selective fibrinolysis and thrombolysis both in vitro and in vivo were achieved, leading to effective vascular recanalization with minimal hemorrhagic risks in mice. Through the combined experimental and computational work, mBiNE@tPA exhibited superior thrombolytic capabilities over formulations with single or no targeting specificity and free tPA. This nanoerythrosome represents a promising platform for targeted therapies.

## Graphical abstract

A multivalent bi‐specific nanoerythrosome (mBiNE@tPA) is developed as a “two‐birds‐one‐stone” platform that simultaneously exploits two principal thrombus components. By integrating erythrocyte membrane‐mediated homologous targeting with cRGD‐based active targeting to activated platelets, mBiNE@tPA achieves enhanced thrombus accumulation, triggered tissue plasminogen activator (tPA) release, and effective vascular recanalization with minimal hemorrhagic risks.

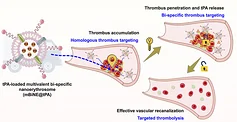

## 1 Introduction

Targeted drug delivery is an effective strategy that can enable precise delivery of drugs to a target site of interest to enhance efficacy and minimize undesirable side effects [1, 2, 3]. Current approaches rely primarily on the use of targeting ligands to exploit certain receptors overexpressed on the diseased cell surface [4, 5]. However, due to the biological barriers and protein corona, the engineered active‐targeting systems may suffer from rapid clearance and off‐target accumulation [6, 7], which can significantly reduce the number of injected nanoparticles that reach the target tissue [8]. Compared to traditional actively targeted nanoparticles, homologous targeting strategies based on cell‐mediated biomimetic systems offer dual advantages. Such systems can not only present “don't eat me” signals to macrophages, thereby prolonging blood circulation time, but also achieve targeted delivery through inherent membrane‐mediated adherence to specific tissues [9, 10]. Unlike conventional active targeting approaches that rely on a single receptor‐ligand interaction, homologous targeting exploits the collective, multivalent interactions conferred by an intact cell membrane. This enables biomimetic nanocarriers to naturally recognize and accumulate at pathological sites composed of their parent cell type. Hence, the development of an approach to designing a targeted nanocarrier with homologous and active targeting to a target tissue represents a significant breakthrough with many potential applications in treatment, diagnosis, and prevention of diseases or disorders.

Plasminogen activator drugs, including tissue plasminogen activator (tPA), urokinase and streptokinase, possess the potential to break up thrombi and restore blood flow for the treatment of thromboembolic diseases such as ischemic stroke, myocardial infarction, pulmonary embolism and deep vein thrombosis [11, 12, 13]. Among them, tPA therapy is especially attractive given that it is a standard treatment approved by the U.S. Food and Drug Administration for acute ischemic stroke. tPA can trigger the conversion of plasminogen into active plasmin through proteolytic cleavage of the Arg561‐Val562 peptide bond, resulting in a fibrinolytic cascade to eliminate thrombi [14, 15]. However, tPA therapy is limited by its very short circulation half‐life (2−6 min) in blood [16] and poor thrombus targeting [17, 18, 19, 20]. Multiple or continuous dosing of tPA is often necessary for thrombolytic treatment, while its systemic off‐target effects lead to a high risk of hemorrhagic complications [21]. Therefore, there is an urgent need to develop a targeted delivery system for more effective and safer thrombolytic therapy [22, 23, 24, 25].

**FIGURE 1:**
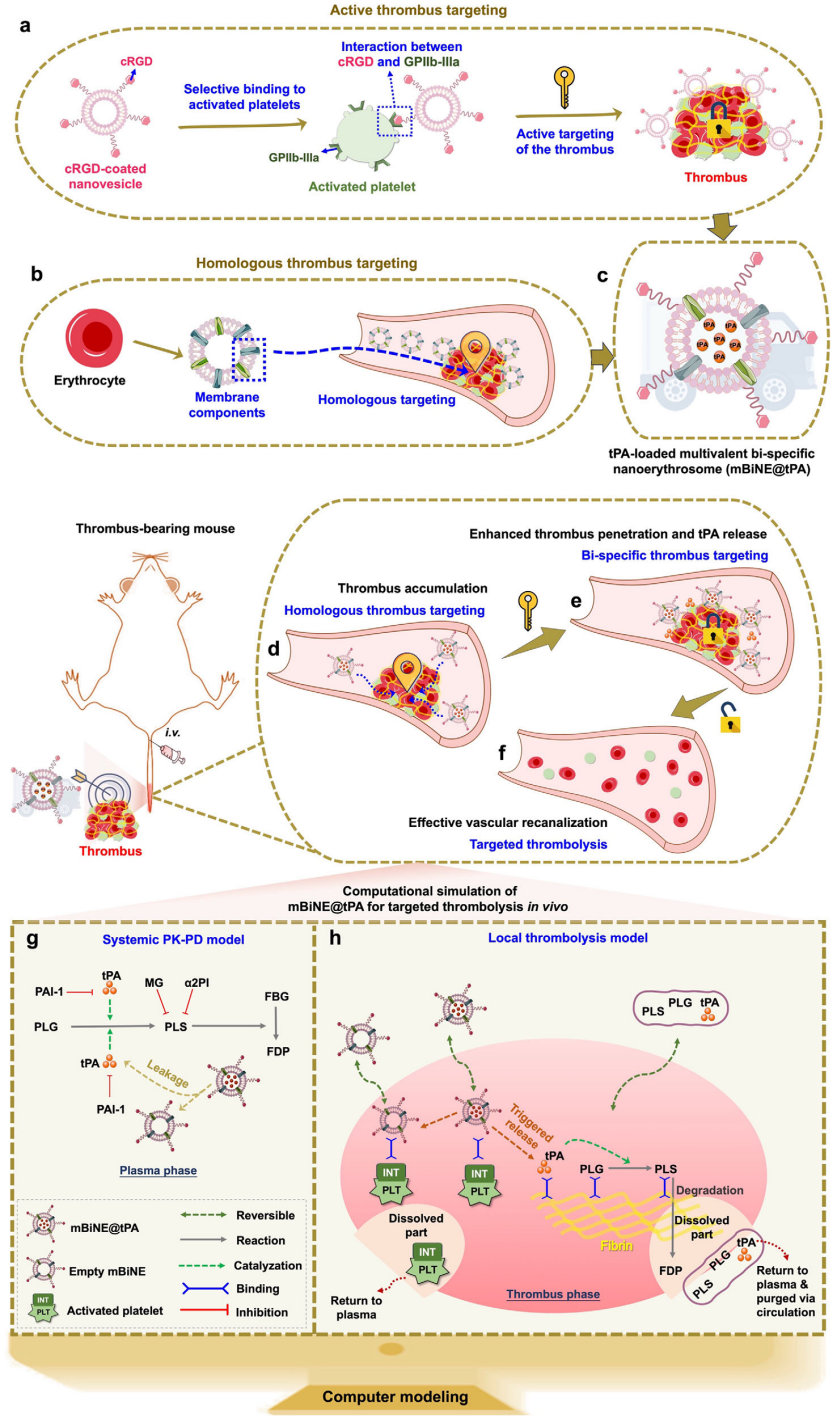
mBiNE@tPA‐based “two‐birds‐one‐stone” approach for targeted thrombolysis. Schematic illustrations of (a) active thrombus targeting and (b) homologous thrombus targeting that are combined to form (c) bi‐specific mBiNE@tPA. Schematic illustrations of (d) homologous targeting and accumulation of mBiNE@tPA at a thrombus site due to homologous thrombus targeting, and (e) enhanced thrombus penetration and triggered tPA release due to the bi‐specific thrombus targeting, leading to (f) targeted thrombolysis and vascular recanalization. Overview of computational simulation of mBiNE@tPA for targeted thrombolysis in vivo, which consists of (g) a compartment systemic PK‐PD model and (h) a local thrombolysis model. PLG, plasminogen; PLS, plasmin; FBG, fibrinogen; FDP, fibrin degradation product; PAI‐1, plasminogen activator inhibitor‐1; MG, α2‐macroglobulin; α2PI, α2 plasmin inhibitor; PLT, activated platelet; INT, GPIIb‐IIIa integrin.

Recent efforts have focused on using targeted synthetic nanocarriers to enhance thrombolytic therapy by chemical conjugation or encapsulation [26, 27, 28, 29, 30, 31]. For example, Korin et al. fabricated shear‐stress‐responsive microaggregates of tPA‐coated poly(lactic‐co‐glycolic) acid (PLGA) nanoparticles to target narrowed or obstructed blood vessels for enhanced thrombolysis [32]. Wang et al. used magnetic nanoparticle‐shelled microbubbles to improve thrombolysis through targeting thrombi under a magnetic field and triggered tPA release upon ultrasound stimulation [33]. However, using the luminal high shear stress or external magnetic field to target thrombi is difficult to implement in a general clinical setting [34]. Inspired by the fibrinogen binding to activated platelets in the process of thrombosis, we have recently coined a “fibrinogen‐mimicking” concept for active thrombus targeting through coating RGD motifs that are present in each of the two Aα chains of fibrinogen (or cRGD with higher binding specificity and affinity) onto the nanovesicle surface [35, 36]. As illustrated in Figure 1a, selective binding of cRGD‐coated nanovesicles to activated platelets was mediated via the interaction between cRGD and GPIIb‐IIIa on the activated platelet surface, thereby enabling active targeting of the thrombus.

Although this active thrombus targeting approach is a notable advancement toward targeted thrombolysis, it lacks the ability of homologous targeting to a thrombus site selectively and may not be sufficient for thrombus anchoring or penetration, especially for red blood cell (RBC)‐rich thrombi wherein activated platelets can be covered by RBCs and become less accessible to the nanocarriers [37].

Recent studies have investigated cell‐mediated delivery approaches using erythrocytes [38], macrophages [39], stem cells [40], and T cells [41]. Among these, utilizing naturally derived nanocarriers from erythrocytes for various drug delivery applications has caught significant attention in the last two decades [42, 43, 44]. However, erythrocyte membrane‐coated nanoparticles have been largely explored for targeted drug delivery in cancer and inflammatory diseases, whereas their application in thrombolytic therapy remains very limited [45, 46]. In the few reported thrombolytic nanosystems employing erythrocyte membranes, the membrane has been employed primarily for immune evasion and prolongation of systemic circulation [47, 48], rather than as a homologous targeting mechanism towards thrombi. Notably, erythrocytes themselves are a key structural component of thrombi and actively participate in thrombus formation through multiple membrane‐mediated interactions, including binding to fibrin and activated platelets via intercellular adhesion molecules and immune proteins on the erythrocyte membrane [49, 50]. Despite this intrinsic thrombus‐tropic nature, erythrocyte membranes have rarely been explored as a homologous targeting interface for thrombus‐directed drug delivery. Meanwhile, platelet membrane‐coated nanoparticles have been investigated for thrombus‐targeted therapy [51, 52]. However, platelet membranes inherently express GPIIb/IIIa, the same target of cRGD, resulting in functional redundancy rather than synergistic targeting. Moreover, activated platelet membranes expose phosphatidylserine, a procoagulant surface that promotes thrombin generation, raising potential safety concerns for thrombolytic applications [53]. In contrast, erythrocyte membranes interact with thrombus constituents via distinct molecular pathways [54, 55], enabling a complementary bi‐specific targeting strategy. Erythrocytes also offer practical advantages, including their high abundance in blood, lack of nuclei and organelles, and the relative simplicity of membrane isolation and scale‐up [44]. To the best of our knowledge, the integration of erythrocyte membrane‐mediated homologous thrombus targeting with ligand‐based active targeting within a single nanoplatform for thrombolysis has not been reported. Inspired by the thrombus‐tropic and camouflagic properties of erythrocytes in blood circulation, we report herein the development of a nanovesicle, termed a nanoerythrosome, that utilizes erythrocyte membranes as a biointerface to enable innate homologous thrombus targeting (Figure 1b). Our design preserves the intact erythrocyte membrane landscape, thereby inheriting the natural, multivalent thrombus‐tropic interactions of erythrocytes through collective biomimetic mechanisms rather than reliance on a single receptor‐ligand pair.

In this article, we report a “two‐birds‐one‐stone” approach that combines homologous and active thrombus targeting for effective targeted thrombolysis. Unlike conventional dual‐targeting strategies, which often employ two targeting ligands to enhance targeting efficiency towards related cellular targets through parallel mechanisms, our “two‐birds‐one‐stone” concept utilizes a single nanoplatform (one stone) to simultaneously exploit two distinct and principal thrombus components (two birds): the thrombus microenvironment via erythrocyte membrane‐mediated homologous targeting, and activated platelets via cRGD‐based active targeting. These two mechanisms operate synergistically. Specifically, the erythrocyte membrane enables broad‐spectrum thrombus docking through multivalent membrane interactions, while cRGD provides molecular‐level anchoring to activated platelets, thereby facilitating localized retention and triggered drug release.

In the study, a tPA‐loaded multivalent bi‐specific nanoerythrosome (Figure 1c), denoted as mBiNE@tPA, is fabricated to exploit two main thrombus components by utilizing the natural erythrocyte membrane for homologous targeting and fibrinogen‐derived cRGD motifs for active targeting. As illustrated in Figure 1d–f, mBiNE@tPA is designed to exhibit homologous targeting and accumulation at a thrombus site specifically, enhanced thrombus penetration, and locally triggered tPA release through its bi‐specific targeting mechanism, ultimately leading to targeted thrombolysis and vascular recanalization. This bio‐inspired system demonstrated favorable pharmacokinetic and biosafety profiles and prolonged circulation time. The bi‐specificity was confirmed under physiological flow conditions in a microfluidic system and in a mouse tail thrombosis model. The triggered tPA release mechanism was elucidated. Efficient and selective fibrinolysis and thrombolysis both in vitro and in vivo were achieved, leading to effective vascular recanalization with minimal hemorrhagic risks in mice. In addition, we further developed a computational model to mimic the triggered tPA release from mBiNE@tPA, and integrated this with a systemic pharmacokinetics‐pharmacodynamics (PK‐PD) model (Figure 1g) and a local thrombolysis model (Figure 1h) to simulate targeted thrombolysis under similar in vitro and in vivo experimental conditions, thereby providing additional mechanistic insights into the transport and actions of mBiNE@tPA at a thrombus. Our combined experimental and computational work demonstrates this bi‐specific nanoerythrosome as a novel platform for more efficient and safer thrombolytic therapy. This strategy presents a new perspective for targeted delivery, unlocking great potential for effective treatment, diagnosis, and prevention of a wide range of diseases or disorders, including cardiovascular diseases, cancer, and infectious diseases.

## 2 Materials and Methods

### 2.1 Materials

Tissue plasminogen activator (tPA, alteplase) was a product of Boehringer Ingelheim (Germany). 1, 2‐distearoyl‐sn‐glycero‐3‐phosphoethanolamine‐N‐(amino(polyethylene glycol)‐2000] (DSPE‐PEG‐NH_2_), cyclo(Arg‐Gly‐Asp‐d‐Phe‐Val) (cRGD), 4‐dimethylaminopyridine (DMAP), N‐hydroxy succinimide (NHS), 1‐(3‐dimethylaminopropyl)‐3‐ethyl carbodiimide hydrochloride (EDC), L‐α‐phosphatidylethanolamine‐N‐(7‐nitro‐2‐1,3‐benzoxadiazol‐4‐yl) (NBD‐PE), L‐α‐phosphatidylethanolamine‐N‐(lissamine rhodamine B sulfonyl) (Rhod‐PE), phosphate‐buffered saline (PBS, pH = 7.4, without calcium or magnesium), fluorescein isothiocyanate (FITC), Triton X‐100, thrombin, plasminogen, fibrinogen, tris(hydroxymethyl) aminomethane, agar, the chromogenic substrate S‐2251 (D‐Val‐Leu‐Lys 4‐nitroanilide dihydrochloride) and ethylene diamine tetraacetic acid (EDTA) were purchased from Sigma‐Aldrich (Dorset, UK). Cy5‑labeled cRGD‑PEG‑DSPE was obtained from Xi'an Ruixi Biological Technology Co., Ltd (Xi'an, China). Centrifugal concentrators were purchased from Fisher Scientific (Loughborough, UK). Whole sheep blood was obtained from TCS Biosciences Ltd (Buckingham, UK). The Avanti mini extruder set, polycarbonate membranes, and filter supports were purchased from Avanti Polar Lipids Inc. (USA). Carrageenan, trypsin, trypsin inhibitor, 3‐(4,5‐dimethylthiazol‐2‐yl)‐2,5‐diphenyltetrazolium bromide (MTT), acid citrate dextrose (ACD), chloroform (CHCl_3_), absolute ethyl alcohol, sodium chloride (NaCl), calcium chloride (CaCl_2_), hydrochloric acid, and sodium hydroxide were obtained from VWR (Lutterworth, UK) and Adamas‐beta (Shanghai, China).

### 2.2 Preparation and Characterization of Erythrocyte Ghosts

Briefly, 2 mL of sheep whole blood (or mouse whole blood for mouse studies) in a 2 mL tube was centrifuged at 3000 rpm (1238 × g) for 5 min at 4°C to remove serum, and the erythrocytes were obtained. Then, the erythrocytes in the tube were washed three times through resuspension in 1 mL PBS buffer (pH 7.4, without calcium or magnesium) followed by centrifugation at 3000 rpm (1238 × g) for 5 min at 4°C. As shown in Figure S1a, the resulting erythrocyte pellet was treated with 1.8 mL of EDTA solution (0.2 mM) to induce membrane rupture and resuspended. Subsequently, the cell solution was adjusted to 2 mL by adding 0.2 mL of 10X PBS (without calcium and magnesium). This hypotonic treatment caused the cells to swell and release their hemoglobin. The resulting solution was centrifuged at 12 100 rpm (20 133 × g) for 15 min at 4°C to remove the supernatant. This process was repeated seven cycles until the supernatant was free of red color. The resulting erythrocyte ghosts were finally re‐suspended in 2 mL of pH 7.4 PBS for storage at 4°C.

The protein content of the erythrocyte ghost membranes was quantified using the bicinchoninic acid (BCA) protein assay kit (Thermo Fisher Scientific). Membrane proteins in the membrane of erythrocyte ghosts were identified by sodium dodecyl sulfate‐polyacrylamide gel electrophoresis (SDS‐PAGE) using a polyacrylamide gel and compared to a pre‐stained protein ladder. Briefly, erythrocyte ghosts were mixed 1:1 with Sample Loading Buffer (2X) and run on a precast NuPAGE 4%–12% Bis‐Tris Protein Gel (Invitrogen) in NuPAGE MOPS SDS Running Buffer (Invitrogen). The gel was run at 150 V for approximately 1 h. The resulting gel was stained overnight using SimplyBlue SafeStain (Invitrogen).

Furthermore, the CD47 and CD235a (glycophorin A) proteins derived from erythrocytes were determined by western blotting analysis. Briefly, all proteins in each sample were separated by SDS‐PAGE, transferred to nitrocellulose membranes, and incubated with primary antibodies against CD47 (Abcam, UK) or CD235a (Proteintech, USA) overnight at 4°C, followed by staining with horseradish peroxidase (HRP)‐conjugated secondary antibodies (Abcam, UK) and observation by the GelDoc‐It310 (UVP, USA) imaging system.

### 2.3 Preparation and Characterization of tPA‐Loaded Multivalent Bi‐Specific Nanoerythrosomes

First, a DSPE‐PEG‐cRGD lipid was synthesized by an amidation reaction between free ‐NH_2_ in DSPE‐PEG‐NH_2_ and free ‐COOH in cRGD peptide according to our established protocol [35, 36]. Subsequently, the obtained DSPE‐PEG‐cRGD lipid (5.0 × 10^−7^ mol) was dissolved in chloroform in a 25‐mL round‐bottom flask. A lipid film was formed by removing the organic solvent via rotary evaporation. Then, 5 mL of the obtained erythrocyte ghosts in PBS solution (2 mg mL^−1^ of proteins) and tPA (5 mg) were added into the flask. The lipid film was hydrated in the mixture at 37°C for 1 h. The tPA‐loaded multivalent bi‐specific nanoerythrosome (mBiNE@tPA) system was obtained following sonication (pulse mode: 5 s on, 5 s off) for 30 min on ice, and serial extrusion (21 times) through a 200‐nm polycarbonate membrane filter in an extruder set (Avanti Polar Lipids Inc.) or a benchtop pressure‐driven extruder (designed and set up in house, Figure S7), followed by removal of the unencapsulated tPA using an ultracentrifugation tube with a MWCO of 100 kDa (Satorius, UK) at 4°C. tPA encapsulation, as opposed to surface adsorption, was confirmed using a trypsin protection assay with S‑2251 chromogenic substrate‐based quantification of tPA activity (Figure S8). During extrusion, sonicated erythrocyte membrane fragments reassembled into closed vesicles, thereby encapsulating a portion of the surrounding aqueous tPA solution within the newly formed lumen [35, 44]. As a hydrophilic protein, tPA remains confined within the aqueous lumen, as confirmed by the trypsin protection assay and the minimal leakage observed.

Liposomal control formulations were prepared using a standard thin‑film hydration method. Briefly, a lipid mixture (20 mg DPPC, 7 mg cholesterol, and 5.0 × 10^−7^ mol DSPE‑PEG‑cRGD) was dissolved in a chloroform/ethyl acetate mixture (3:2, v/v) in a round‐bottom flask to ensure molecular‐level homogeneity. Following solvent evaporation using a rotary evaporator (40°C, 120 rpm), a thin lipid film formed on the interior of the flask. The film was subsequently hydrated with 5 mL PBS (pH 7.4) containing tPA (5 mg) under continuous stirring at 37°C for 1 h. The tPA‐loaded liposomes were obtained by sonication (pulse mode: 5 s on, 5 s off) for 30 min on ice, followed by extrusion through a 200‐nm polycarbonate membrane filter using an extruder set (Avanti Polar Lipids Inc.). Unencapsulated tPA was removed by ultracentrifugation (100 kDa MWCO).

The enzymatic activity of tPA in the resulting filtrate and retentate was measured separately using the chromogenic substrate S‐2251 assay to confirm the effective purification. The tPA content in the nanoerythrosomes was determined by the chromogenic substrate S‐2251 assay to calculate the encapsulation efficiency (EE) and loading capacity (LC) according to the following equations:

(1) $$\mathrm{EE}\;\left(\%\right)=\frac{m_{l}}{m_{t}}\times 100$$

where *m_l_* (mg) is the weight of loaded tPA and *m_t_* (mg) is the total weight of tPA in the initial loading solution.

(2) $$\mathrm{Loading}\;\mathrm{capacity}\;\left(\mathrm{LC}\right)=\frac{\mathrm{Encapsulated}\;\mathrm{tPA}\;\mathrm{mass}\;\left(\mu g\right)}{\mathrm{Membrane}\;\mathrm{protein}\;\mathrm{mass}\;\left(\mathrm{mg}\right)}$$

The hydrodynamic size and PDI of mBiNE@tPA were measured by DLS (Zetasizer Nano S, Malvern, UK). Its morphology was visualized by a 120 kV Tecnai 12 cryo‐electron microscopy (cryo‐EM), which was equipped with a TVIPS XF416 4K CMOS camera. Its zeta potential was measured by a zeta potential analyzer (Zetasizer Lab, Malvern, UK). The tPA‐loaded nanoerythrosome without cRGD coating (NE@tPA), as well as empty mBiNE and NE, were synthesized and characterized for comparison. The nanoparticle concentration of mBiNE@tPA was determined by nanoparticle tracking analysis (NTA) using a NanoSight NS300 system (Malvern, UK) configured with a 532 nm laser, which provides a good scattering signal while minimizing background fluorescence. The samples were suspended in PBS buffer and measured 3 times for 30 s each using an O‐ring chamber.

The cRGD surface density on mBiNE@tPA was determined using a fluorescence‑based quantification assay performed on a microplate reader (Tecan, Switzerland), with excitation and emission wavelengths set at 645 nm and 670 nm, respectively. Briefly, a fluorescently labeled cRGD lipid (Cy5‑cRGD‑PEG‑DSPE) was incorporated into mBiNE@tPA at the same molar ratio as in the non‑labeled formulation. After purification, the fluorescence intensity of the resulting mBiNE@tPA was quantified based on a standard calibration curve generated using Cy5‑cRGD‑PEG‑DSPE. The cRGD surface density (3.97 × 10^4^ cRGD molecules per particle) was calculated based on the measured particle concentration obtained from NTA and the quantified concentration of Cy5‑cRGD‑PEG‑DSPE (Figure S14c).

### 2.4 Thrombus Targeting in a Microfluidics System

Flow chambers with Vena8 Fluoro+ BioChip (length, 2.8 cm; width, 0.04 cm; and height, 0.01 cm; Cellix Ltd., Ireland) were coated with fibrillar collagen (150 mg mL^−1^) at 4°C overnight. Channels were then treated with 1 wt% bovine serum albumin in HT buffer (134 mM NaCl, 2.9 mM KCl, 1 mM MgCl_2_, 0.34 mM Na_2_HPO_4_, 12 mM NaHCO_3_, 20 mM Hepes, and 5 mM glucose (pH 7.3)] at room temperature for 30 min and washed with HT buffer prior perfusion. Healthy SD rats were anesthetized by intraperitoneal injection with sodium pentobarbital (50 mg kg^−1^). Whole blood was taken from the abdominal aorta and collected in a tube containing sodium citrate as an anticoagulant (volume ratio of blood to citrate at 1:9). Nonocclusive thrombi were formed through perfusion of the citrated blood in the collagen‐coated channels at a wall shear rate of 1000 s^−1^ at room temperature. The channels were then washed with HT buffer by perfusion for 2 min. The tPA‐loaded liposome without cRGD coating (denoted as Lip@tPA), cRGD‐coated, tPA‐loaded liposome (denoted as cRGD‐Lip@tPA), and NE@tPA were used as controls. In this study, FITC‐labeled Lip@tPA, cRGD‐Lip@tPA, NE@tPA or mBiNE@tPA (equivalent tPA dose of 0.1 mg mL^−1^) was perfused in the thrombi‐bearing channels for 5 min to demonstrate thrombus targeting activity. The channels were washed by perfusion with HT buffer for 2 min and imaged using a NIB910 (Nexcope, China) fluorescence microscope equipped with a C11440‐42U30 CMOS camera (Hamamatsu Photonics K.K., Japan).

### 2.5 Animal Experiments

All animal experiments were performed in strict agreement with the Principles of Animal Care and Use, with ethical approval obtained from the Animal Research Committee of Shanghai Jiao Tong University School of Medicine Affiliated Sixth People's Hospital (ethical approval number: 2022‐0188). Healthy male Sprague–Dawley (SD) rats (∼220 g) and Kunming (KM) mice (∼22 g) were purchased from Shanghai SLAC Laboratory Animal Co., Ltd.

### 2.6 Mouse Tail Thrombosis Model

KM mice were starved for 8 h before treatment via intraperitoneal injection with fresh carrageenan saline solution at a dose of 20 mg kg^−1^. The mice were then fed at 20°C, and after 24 h the black‐tail thrombus could be observed. According to the established protocol [56, 57], to evaluate the in vivo targeting and thrombolytic efficacy, all formulations were administered via intravenous injection in a healthy segment of the tail vein proximal to the body, instead of via local injection at the thrombus site. This administration route ensured that the formulations would circulate systemically before reaching the distal thrombus, thereby providing a valid assessment of their targeting capability.

### 2.7 Measurement of Blood Flow

Tail thrombus‐bearing KM mice were anesthetized via intraperitoneal injection with sodium pentobarbital at a dose of 50 mg kg^−1^. A laser speckle contrast imager (LSCI) with a PeriCam PSI HR system (Perimed, Sweden) was employed to measure the mean blood flow intensity in the tail. Images of blood flow in tails were obtained, and the mean blood flow intensity in the regions of interest (ROIs) was analyzed by PIMSoft software.

### 2.8 In Vivo Thrombolysis

KM mice with a similar black‐tail thrombus length were randomly divided into seven groups in a blind study and intravenously injected with a tPA‐containing sample at an equivalent tPA dose of 10 mg kg^−1^, such as mBiNE@tPA, NE@tPA, free tPA, Lip@tPA and cRGD‐Lip@tPA, or a negative control sample, such as empty mBiNE and pH 7.4 PBS buffer. The tail thrombus lengths before and after treatment were recorded, and the bloodstream recovery was assessed. The body weight of each animal was recorded daily. After 7 days post‐injection, all animals were sacrificed after anesthetization via intraperitoneal injection with sodium pentobarbital (150 mg kg^−1^), and their tails in the ROI were collected and stored in 4% (w/v) paraformaldehyde solution at 4°C for 48 h. After decalcification, the tails were embedded in paraffin and cut into small slices. Those slices were then stained with hematoxylin and eosin (H&E) for histochemical analysis.

### 2.9 Computational Model

A computational model was developed as a mechanistic tool to elucidate experimental observations through an integrated approach. Model parameters were first calibrated against in vitro release kinetics and then validated against independent experimental results, including both in vitro halo clot lysis and in vivo thrombolysis. This validated framework subsequently provided detailed mechanistic insights into the transport and actions of mBiNE@tPA at a thrombus.

### 2.10 Computational Simulation of Triggered tPA Release and Thrombolysis In Vitro

The computational method for in vitro testing followed the methodologies established in our previous work [35]. Briefly, computational simulations mimicked the tPA release experiments to corroborate the proposed mechanism of triggered release. The key reaction kinetic parameters were estimated and applied to the tPA release prediction and thrombolysis simulation. The mBiNE@tPA system was assumed to be homogeneously mixed and mathematically represented through a combination of ordinary differential equations and algebraic equations. Further information about the reaction kinetics and the specific parameters used can be found in our earlier publication [35]. To solve these model equations, we employed the ode15s solver, which is a variable‐order method integrated into MATLAB R2023a.

### 2.11 Computational Simulation of Targeted Thrombolysis In Vivo

A systemic PK‐PD model, in combination with a local mouse tail thrombolysis model, was developed for targeted thrombolysis by mBiNE@tPA. The PK‐PD model describes the plasma level of species in the mouse body, and the solution of the PK‐PD model provides the inlet boundary condition for the local thrombolysis model. The temporal concentration profiles of species were simulated by a one‐compartment model.

For free tPA administration:

(3) $$\frac{dC_{\mathrm{tPA},\mathrm{sys}}}{\mathrm{dt}}=\frac{I}{V_{d,\mathrm{tPA}}M_{w,\mathrm{tPA}}\;}\;-\;k_{\mathrm{el},\mathrm{tPA}}C_{\mathrm{tPA},\;\mathrm{sys}}+S_{\mathrm{tPA}}{+R}_{\mathrm{tPA}}^{\mathrm{plasma}}$$

For mBiNE@tPA administration:

(4) $$\begin{array}{ccc} \frac{dC_{\mathrm{mBiNE}@\mathrm{tPA},\mathrm{sys}}}{\mathrm{dt}} & = & \frac{I}{V_{d,\mathrm{mBiNE}@\mathrm{tPA}}M_{w,\mathrm{mBiNE}@\mathrm{tPA}}\;}\\ - & & k_{\mathrm{el},\mathrm{mBiNE}@\mathrm{tPA}}C_{\mathrm{mBiNE}@\mathrm{tPA},\mathrm{sys}}{\;+\;R}_{\mathrm{mBiNE}@\mathrm{tPA}}^{\mathrm{plasma}} \end{array}$$

For tPA leaked mBiNE@tPA:

(5) $$\frac{dC_{\mathrm{mBiNE},\mathrm{sys}}}{\mathrm{dt}}=-k_{\mathrm{el},\mathrm{mBiNE}@\mathrm{tPA}}C_{\mathrm{mBiNE},\mathrm{sys}}{\;+\;R}_{\mathrm{mBiNE}}^{\mathrm{plasma}}$$

For other fibrinolytic proteins:

(6) $$\begin{array}{ccc} & & \frac{dC_{i,\mathrm{sys}}}{\mathrm{dt}}=-k_{el_{i}}C_{i,\mathrm{sys}}{+R}_{i}^{\mathrm{plasma}}+S_{i},\\ & & \;i=\mathrm{PLG},\;\mathrm{PLS},\;\mathrm{AP},\;\mathrm{FBG},\;\mathrm{MG}\;\mathrm{PAI}-1\;\mathrm{and}\;\mathrm{AP}-\mathrm{PLS} \end{array}$$

where PLG is plasminogen, PLS is plasmin, AP is α_2_‐antiplasmin, FBG is fibrinogen, MG is α_2_‐macroglobulin, PAI‐1 is plasminogen activator inhibitor‐1, AP‐PLS is α_2_‐antiplasmin‐plasmin complex, *V*_*d*, *mBiNE*@*tPA* _ is a distribution volume in the central compartment, *M*_*w*, *i*_ is a molecular weight, *k*_*el*, *i*_ is an elimination constant, *C*_*i*, *sys*_ is a systemic concentration, *S*_*i* _is the secretion of tPA or other components, and $R_{i}^{Plasma}$ represents a fibrinolysis reaction in the plasma. The clearance (*k*_*el*, *i*_) of mBiNE@tPA is assumed to be the same as that of empty mBiNE. Detailed calculations of the pharmacokinetic parameters and reactions in the source terms can be found in Sections S2.1 and S2.2.

Mathematical modeling of the mouse tail thrombolysis involves solving a set of convection‐diffusion‐reaction differential equations for each species. The fibrinolytic and triggered tPA release reactions, as shown in the reaction kinetic section 2.1 in the Supporting Information, were treated as source terms in these differential equations. All the transport equations were implemented into the customized solvers developed using OpenFOAM 9.0 software. By assuming all the space in the mouse tail is thrombosed except the coccyx, we built an ideal concentric cone geometry to represent the mouse tail. The thrombus within the mouse tail is described as a porous structure, consisting of fibrin fibers, activated platelets, and other blood cells. Fibrin fibers and activated platelets are represented in the model with an initial volume fraction of *Φ_f,0_* and *Φ_p,0_*, respectively. Further computational details can be found in the Sections S2.3 and S2.4.

### 2.12 Statistical Analysis

All the results were shown as the average ± standard deviation (SD) (*n* ≥ 3) and plotted by using GraphPad Prism (version 8.0), unless otherwise indicated. Differences between two groups were analyzed for statistical significance using the unpaired Student's *t*‐test, and comparisons among multiple groups were assessed for statistical significance using the one‐way analysis of variance (ANOVA) test. Differences were considered to be statistically significant when the *p*‐value was < 0.05.

## 3 Results and Discussion

### 3.1 Scalable Production and Characterization of mBiNE@Tpa

**FIGURE 2:**
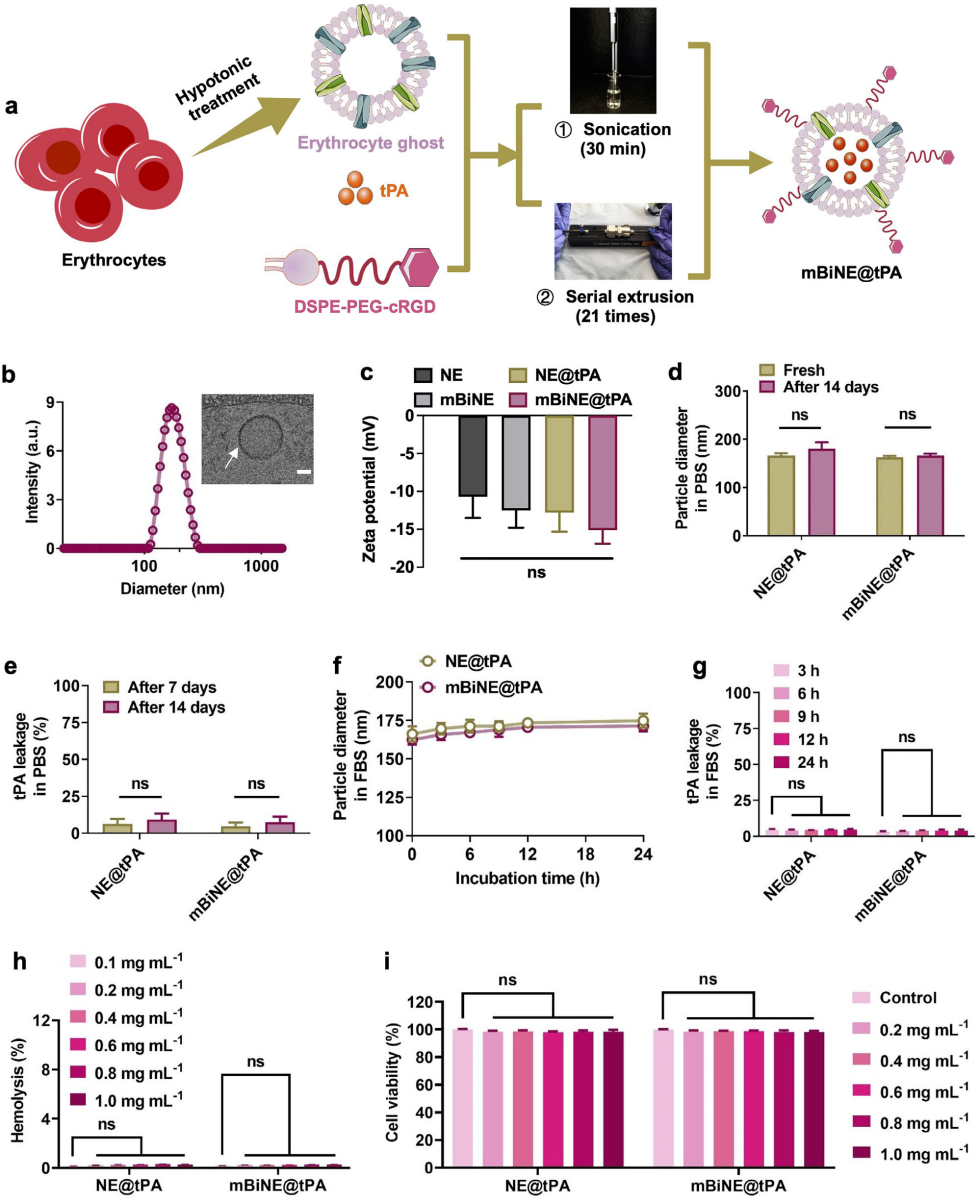
Preparation and characterization of mBiNE@tPA. (a) Schematic illustration of the preparation process of mBiNE@tPA. (b) Typical DLS plot of mBiNE@tPA in pH 7.4 PBS buffer. Inset: its representative cryo‐TEM image. Scale bar, 50 nm. (c) Zeta potentials of NE, mBiNE, NE@tPA and mBiNE@tPA. (d) Particle diameters of NE@tPA and mBiNE@tPA before and after storage in pH 7.4 PBS buffer at 4°C for 14 days. (e) tPA leakage profiles of NE@tPA and mBiNE@tPA after storage in pH 7.4 PBS buffer at 4°C for 7 and 14 days. (f‐g) Variations in particle diameter and tPA leakage of NE@tPA and mBiNE@tPA in 50% (v/v) FBS solution in pH 7.4 PBS buffer during 24 h of incubation in a water bath at 37°C under shaking at 120 rpm. (h) Hemolytic activities of NE@tPA and mBiNE@tPA as a function of tPA concentration after 1 h of incubation in pH 7.4 PBS buffer at 37°C. (i) HUVEC viabilities after incubation with NE@tPA and mBiNE@tPA at various tPA concentrations for 24 h, as measured by MTT assay. HUVECs in medium only were used as a control. Data are presented as the average ± SD (*n* = 3). Statistical analysis was performed by the ANOVA test (ns, not significant).

Erythrocyte ghosts were obtained through hypotonic treatment (Figure S1a). The multivalent bi‐specific nanoerythrosomes, mBiNE@tPA, were fabricated by sonication followed by serial extrusion of the mixture of erythrocyte ghosts, DSPE‐PEG‐cRGD, and tPA through a 200‐nm polycarbonate membrane, as depicted in Figure 2a. The tPA‐loaded nanoerythrosomes without cRGD coating (denoted as NE@tPA) were constructed for comparison. The SDS‐PAGE result shown in Figure S1b suggested that all the proteins associated with the erythrocyte membranes, as listed in Figure S1c [58], were retained in the erythrocyte ghosts after hypotonic swelling of erythrocytes. A key membrane protein that is crucial for ensuring evasion from the immune system is the self‐identifying protein CD47, with a reported molecular weight between 50 and 60 kDa [59]. One of the bands between 50 and 60 kDa in Figure S1b appeared to be present at a lower concentration than some of the other membrane proteins of the erythrocyte ghosts, but it could still be detected.

The retention of key erythrocyte membrane proteins during fabrication of mBiNE@tPA was confirmed by western blotting analysis. As shown in Figures S2 and S3, the disappearance of 𝛽‐actin bands in western blotting indicates that during extraction of erythrocyte membranes, erythrocytes were adequately ruptured, leading to the release of their contents. Critically, both CD47 and CD235a (glycophorin A) were successfully retained, confirming the preservation of a native membrane interface on the nanoerythrosomes. CD47 fulfills its canonical role as a “don't eat me” signal by engaging signal regulatory protein α (SIRPα) on phagocytes, which is essential for minimizing immune clearance and achieving prolonged circulation [60, 61], a fundamental prerequisite for targeted delivery. Beyond this, as part of the intact membrane's complex landscape, erythrocyte CD47 may participate in broader interactions within the thrombotic microenvironment, such as with thrombospondin‐1 (TSP‐1) and fibrinogen [62, 63]. Similarly, CD235a is not merely a marker but is indicative of a functional erythrocyte‐derived surface. It is recognized that growing thrombi release increased levels of CD235a^+^ erythrocyte‐derived microparticles into the circulation, identifying them as potential biomarkers of ongoing thrombosis [64]. This probably supports the rationale that the intact erythrocyte membrane is inherently predisposed to engage with the thrombotic niche. Therefore, the homologous targeting of mBiNE@tPA stems from multivalent interactions enabled by this intact membrane cloak as a holistic biointerface. It leverages a repertoire of potential interactions with various cellular and proteinaceous components (e.g., endothelial cells, platelets, fibrin(ogen), and released proteins) [54, 55, 65], rather than relying on a single receptor‐ligand pair. This multivalent, biomimetic foundation synergizes with the cRGD‐mediated active targeting to achieve robust thrombus accumulation.

As shown in Figures 2b and S4, mBiNE@tPA was monodisperse, with a dynamic light scattering (DLS) size and polydispersity index (PDI) of 162.4 ± 3.1 nm and 0.213 ± 0.011, respectively. These values were comparable to those of NE@tPA (166.1 ± 4.9 nm, PDI = 0.234 ± 0.014), mBiNE (158.6 ± 5.2 nm, PDI = 0.225 ± 0.026), and NE (159.7 ± 5.8 nm, PDI = 0.209 ± 0.022). The cryo‐transmission electron microscopy (cryo‐TEM) image shown in the inset of Figure 2b revealed that mBiNE@tPA exhibited a spherical shape (white arrow), with a TEM particle size consistent with the corresponding DLS result. Figure 2c shows that mBiNE@tPA exhibited a zeta potential of ‐15.1 ± 1.8 mV, attributed to the negatively charged erythrocyte membrane. No significant differences were observed when compared with NE@tPA (‐12.8 ± 2.5 mV), mBiNE (‐12.5 ± 2.3 mV) or NE (‐10.7 ± 2.8 mV). Figure S5 shows that the encapsulation efficiency and loading capacity of mBiNE@tPA (EE = 29.2 ± 2.5%; LC = 146.0 ± 12.5 µg tPA mg^−1^ protein) were comparable to those of NE@tPA (EE = 25.3 ± 3.2%; LC = 126.5 ± 16.0 µg tPA mg^−1^ protein).

Good stability of mBiNE@tPA and NE@tPA was evidenced by the non‐significant particle size change and negligible tPA leakage after 14 days of storage in pH 7.4 PBS buffer at 4°C (Figure 2d,e). The observed minimal tPA leakage indicates the high integrity of the erythrocyte membrane shell, consistent with the reported stability of erythrocyte‐derived vesicles [44]. Their serum stability at 37°C under shaking at 120 rpm over 24 h was further confirmed, with no significant changes in particle size, tPA leakage or zeta potential (Figures 2f,g and S6). The favorable stability profiles could be attributed to their negative surface charge [66]. Their good biocompatibility in vitro was also demonstrated by negligible hemolysis and high tolerance toward human umbilical vascular endothelial cells (HUVEC), as shown in Figure 2h,i.

The scalable production of mBiNE@tPA was demonstrated using our established method based on a benchtop pressure‐driven extruder, as shown in Figure S7. Compared with the use of an Avanti mini extruder, this nitrogen‐driven extruder could allow the production at scale of mBiNE@tPA with uniform product quality, increasing the batch size by 50‐folds while considerably reducing the sample loss during the extrusion process and the time required for production.

### 3.2 Active Thrombus Targeting for Triggered tPA Release and Targeted Thrombolysis In Vitro

**FIGURE 3:**
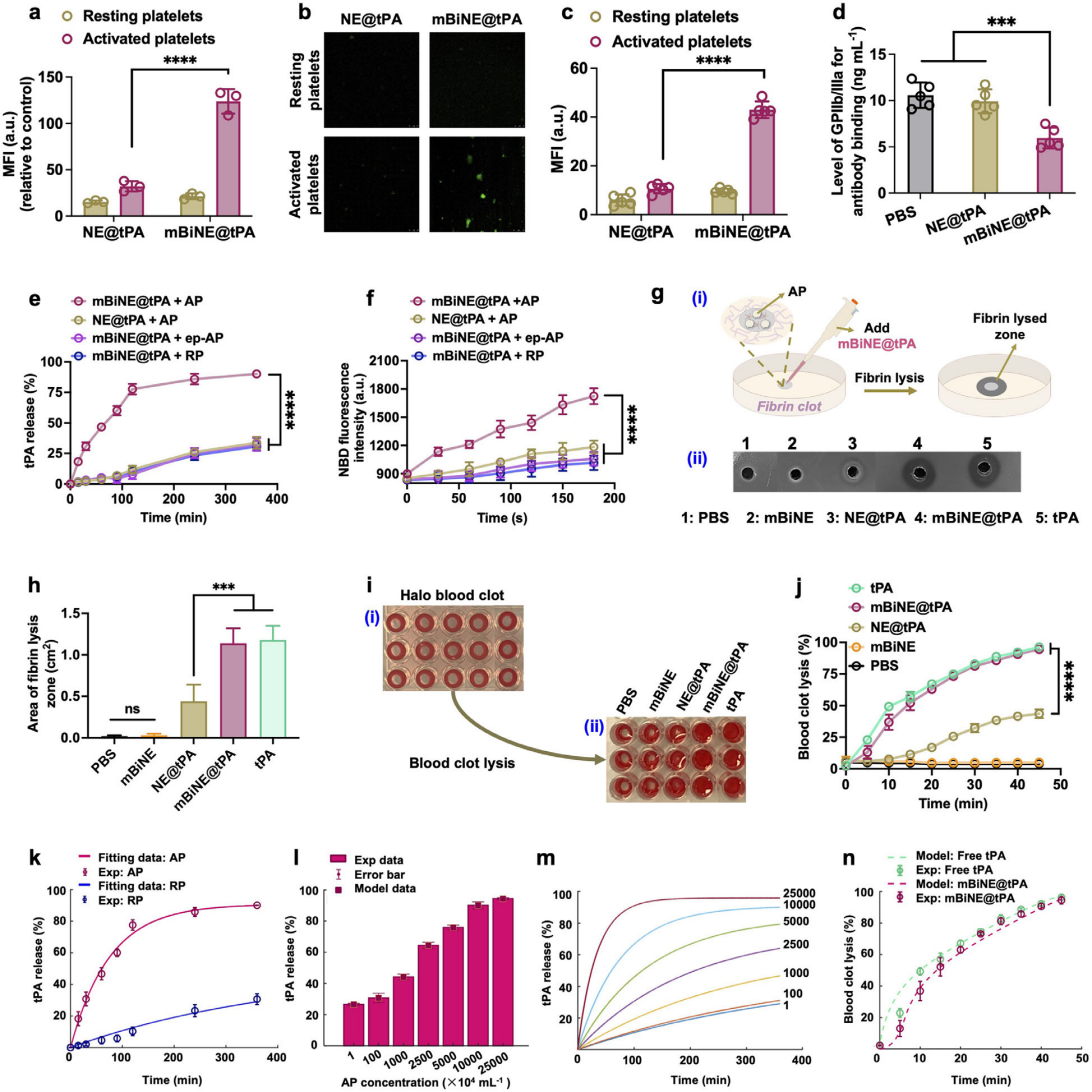
Active thrombus targeting for triggered tPA release and targeted thrombolysis in vitro via experiments and model predictions. (a) Flow cytometric mean fluorescence intensities (MFI) of resting or activated platelets treated with FITC‐labeled NE@tPA and mBiNE@tPA (equivalent tPA dose of 0.2 mg mL^−1^) at 37°C for 4 h, respectively, relative to control treated with pH 7.4 PBS. (b) CLSM images of resting and activated platelets incubated with FITC‐labeled NE@tPA and mBiNE@tPA (equivalent tPA dose of 0.2 mg mL^−1^) at 37°C for 4 h, respectively. Scale bar, 50 µm. (c) MFI in the CLSM images, shown in panel (b), of resting or activated platelets after incubation with FITC‐labeled NE@tPA and mBiNE@tPA (equivalent tPA dose of 0.2 mg mL^−1^) at 37°C for 4 h, respectively, as analyzed by ImageJ. (d) Level of GPIIb‐IIIa for antibody binding on activated platelets treated with pH 7.4 PBS buffer, NE@tPA and mBiNE@tPA (equivalent tPA dose of 0.2 mg mL^−1^) at 37°C for 10 min, respectively. (e) tPA release profiles of NE@tPA and mBiNE@tPA (equivalent tPA dose of 0.5 mg mL^−1^) and (f) real‐time monitoring of NBD fluorescence intensities of mBiNE@tPA coated with both NBD (1 mol%) and Rhod (1 mol%) incubated with 1.0 × 10^8^ mL^−1^ resting platelets (RP) or activated platelets (AP) or eptifibatide (100 µg mL^−1^) pre‐treated activated platelets (ep‐AP). (g) Schematic illustration and representative photograph of fibrin clots, and (h) calculated areas of fibrin clot lysis zones after treatment with pH 7.4 PBS buffer, mBiNE, NE@tPA, mBiNE@tPA and free tPA (equivalent tPA dose of 1 mg mL^−1^), respectively, in the presence of 1.0 × 10^8^ mL^−1^ activated platelets at 37°C for 6 h. (i) Representative photographs of halo blood clots before and after treatment with pH 7.4 PBS buffer, mBiNE, NE@tPA, mBiNE@tPA and free tPA (equivalent tPA dose of 1 mg mL^−1^), respectively, at 37°C for 45 min. (j) Time‐dependent clot lysis in the halo blood clot model after treatment at 37°C (equivalent tPA dose of 1 mg mL^−1^). Data are presented as the average ± SD (n ≥ 3). Statistical analysis was performed by the ANOVA test (***p < 0.001, ****p < 0.0001, and ns, not significant). Comparison of model predictions with experimental results of tPA release from mBiNE@tPA (k) after 6 h of incubation with 1 × 10^8^ mL^−1^ activated platelets (AP) or resting platelets (RP) or (l) with different concentrations of activated platelets. (m) Model predictions of tPA release from mBiNE@tPA at different concentrations of activated platelets (× 10^4^ mL^−1^) as a function of incubation time. (n) Comparison of model predictions and experimental results of clot lysis in the halo blood clot model treated with mBiNE@tPA and free tPA at 37°C, respectively.

Flow cytometry analysis in Figures 3a and S9–S11 showed that only a low normalized fluorescence intensity relative to the PBS control group was observed for activated platelets treated with FITC‐labeled tPA or NE@tPA, or for resting platelets treated with FITC‐labeled tPA, NE@tPA or mBiNE@tPA. By contrast, FITC‐labeled mBiNE@tPA bound more avidly to activated platelets as compared to resting platelets, showing an over 4‐fold fluorescence enhancement. Confocal laser scanning microscopy (CLSM) imaging analysis in Figure 3b,c further confirmed the selective binding of mBiNE@tPA to activated platelets through active targeting. The cRGD density on mBiNE@tPA could be regulated to manipulate its binding affinity to activated platelets, reaching a plateau at about 4.0 × 10^4^ cRGD per nanoerythrosome (Figure S12). The enzyme‐linked immunosorbent assay (ELISA) measurements showed that treatment of activated platelets with mBiNE@tPA caused a significant reduction in detectable GPIIb‐IIIa integrins, while no significant difference was observed between the NE@tPA and PBS treatment groups (Figure 3d). This indicated that GPIIb‐IIIa on activated platelets could be selectively bound and blocked by cRGD on mBiNE@tPA [67, 68].

tPA release profiles in Figure 3e show that, upon binding of mBiNE@tPA to activated platelets, approximately 77% tPA was rapidly released within 2 h, followed by continued release at a slower rate to reach approximately 90% in 6 h. However, after inhibition of GPIIb‐IIIa integrins with eptifibatide, tPA release was substantially reduced to a level comparable to that achieved by mBiNE@tPA in the presence of resting platelets or by NE@tPA in the presence of activated platelets, reaching approximately 31% within 6 h. These results indicated that tPA release from mBiNE@tPA was triggered by its active targeting to activated platelets through selective binding of cRGD with GPIIb‐IIIa integrins, which was further validated by the activated platelet concentration‐dependent tPA release as shown in Figure S13.

To further elucidate the tPA release mechanism, a fluorescence resonance energy transfer (FRET) assay was conducted using the mBiNE@tPA coated with both FRET donor 7‐nitro‐2‐1,3‐benzoxadiazol‐4‐yl (NBD) and acceptor rhodamine (Rhod). As shown in Figure 3f, incubation of mBiNE@tPA with activated platelets (AP) resulted in a rapid increase in NBD fluorescence intensity, which was attributed to the increased distance between the donor NBD and the acceptor Rhod upon binding of the nanoerythrosome to the activated platelet surface. By comparison, inhibition of GPIIb‐IIIa integrins on activated platelets with eptifibatide (ep‐AP) caused a significant decrease in NBD fluorescence intensity to a level comparable to two controls (mBiNE@tPA + resting platelets (RP), and NE@tPA + activated platelets (AP)). The results suggest that the triggered tPA release profile (Figures 3e and S13) was regulated by the membrane alteration as a result of active targeting of mBiNE@tPA to activated platelets through the specific interaction of cRGD with GPIIb‐IIIa. This is consistent with the previous report by other researchers that the activated‐platelet‐induced responsive drug release was due to membrane destabilization caused by the interaction of targeting process and the mechanical forces generated by this targeting interaction [69, 70, 71].

The selective fibrinolytic activity of mBiNE@tPA was evaluated in the presence of activated platelets using a fibrin agar plate assay (Figure 3g(i)). As shown in Figure 3g(ii),h, only a small fibrinolysis ring around the sample well was observed for the NE@tPA group (0.44 ± 0.23 cm^2^), while there was no notable fibrinolysis after incubation with mBiNE (0.03 ± 0.02 cm^2^) or PBS buffer (0.02 ± 0.01 cm^2^). By contrast, treatment with mBiNE@tPA caused a significantly larger fibrin lysis zone of 1.14 ± 0.18 cm^2^, similar to that caused by free tPA (1.18 ± 0.17 cm^2^).

Further, the targeted thrombolytic activity of mBiNE@tPA was examined in a halo blood clot model (Figure 3i(i)). Figure 3i(ii),j showed almost complete clot dissolution and erythrocyte release after 45 min of incubation with mBiNE@tPA and free tPA, respectively. Time‐dependent quantitative analysis shown in Figure 3j suggested that the thrombolytic rate of mBiNE@tPA was a bit lower than that of free tPA within the initial 10 min, which could be ascribed to the time required for tPA release. By comparison, after 45 min of incubation, NE@tPA without cRGD coating caused a significantly lower level of thrombolysis (∼45%) than mBiNE@tPA and free tPA, while the control groups, mBiNE and PBS buffer, showed negligible thrombolysis (less than 5%).

### 3.3 Computational Simulation of tPA Release and Targeted Thrombolysis In Vitro

A custom‐designed computational model for mBiNE@tPA was initially utilized to simulate tPA release in response to activated platelets, with model parameters being established by fitting our simulation results to experimental data acquired in vitro. Details of the reaction mechanisms can be found in Section S2.1. The initial condition of the model exactly matched the experimental procedure. The stoichiometric coefficient *ν_rel_* in Equations S19 and S21 was fixed at 3394, reflecting the relative number of tPA in mBiNE@tPA, as determined via nanoparticle tracking analysis (NTA) shown in Figure S14a. Figure 3k contrasts the experimental tPA release data in the presence of resting or activated platelets (PLT) with our model's predictions. By analyzing the tPA release data with resting platelets at 1 × 10^8^ mL^−1^ (blue circles in Figure 3k), we estimated the tPA leakage rate constant, *k_leak_*_,_ to be 5.93×10^−9^ s^−1^. Given the leakage rate constant, the binding (*k_a,mBiNE@tPA_*) and unbinding rate constants (*k_d,mBiNE@tPA_*) between mBiNE@tPA and GPIIb‐IIIa integrins (INTs), as well as the release rate constant (*k_rel_*), can be estimated by fitting the simulation results to the tPA release profile in the presence of activated platelets at 1 × 10^8^ mL^−1^ (red circles in Figure 3k). The respective values for these parameters are *k_a,mBiNE@tPA_* = 1.38×10^−2^ µM^−1^ s^−1^, *k_d,mBiNE@tPA_* = 7.59 ×10^−4^ s^−1^, and *k_rel_* = 2.05×10^−1^ s^−1^. Notably, the binding rate of mBiNE@tPA to activated platelets (*k_a,mBiNE@tPA_*) was about 2 times faster than that of the cRGD‐coated, tPA‐loaded liposomes we previously developed [35], while its unbinding rate (*k_d, mBiNE@tPA_*) was 10 times slower, highlighting the superior affinity of mBiNE@tPA for activated platelets. Applying these estimated parameters, the model successfully predicted the amount of tPA released at various activated platelet concentrations. Figure 3l demonstrates a close match between our model's predictions and experimental results across a range of activated platelet concentrations. Figure 3m reveals that within a 6‐h period, the tPA release pattern was very similar at low concentrations of activated platelets, with the tPA release increasing almost linearly over time. At high activated platelet concentrations, the release profile showed a more rapid initial increase and reached its peak more quickly than at lower concentrations. The in vitro thrombolysis model was further validated by the halo blood clot experimental results. As shown in Figure 3n, the predicted extent of lysis with free tPA and mBiNE@tPA agreed well with the experimental data.

### 3.4 Bi‐Specific Thrombus Targeting for Enhanced Accumulation and Penetration at a Thrombus Site in Physiological Flow Conditions and Animals

**FIGURE 4:**
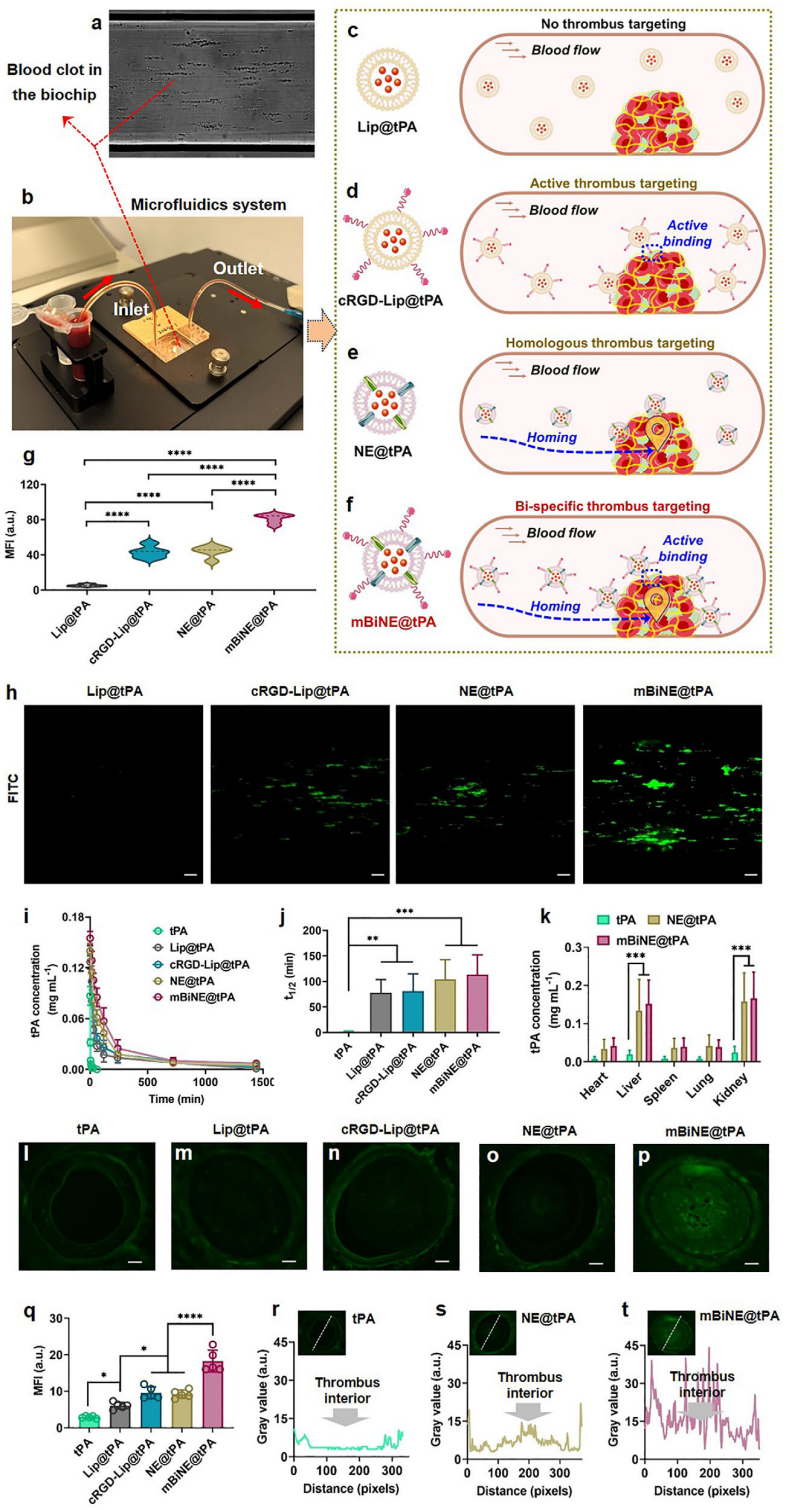
Combination of homologous and active thrombus targeting for enhanced thrombus accumulation and penetration in physiological flow conditions and animals. (a) Representative bright‐field image of blood clots in a flow chamber in the biochip of (b) a microfluidics system setup for monitoring of thrombi under physiological flow conditions by fluorescence microscopy. (c‐f) Schematic illustrations of perfusion with Lip@tPA, cRGD‐Lip@tPA, NE@tPA, and mBiNE@tPA, respectively, in the flow chamber, and the corresponding thrombus targeting mode. (g) MFI analysis by Fiji and (h) representative fluorescence images of blood clots in the flow chamber after perfusion with FITC‐labeled Lip@tPA, cRGD‐Lip@tPA, NE@tPA, and mBiNE@tPA (equivalent tPA dose of 0.1 mg mL^−1^) for 5 min, respectively. Scale bar, 50 µm. (i) Pharmacokinetic profiles, (j) half‐lives (t_1/2_) and (k) tissue distribution after intravenous injection of healthy SD rats with FITC‐labeled free tPA, FITC‐labeled Lip@tPA, cRGD‐Lip@tPA, NE@tPA and mBiNE@tPA (equivalent tPA dose of 10 mg kg^−1^), respectively. (l‐p) Fluorescence images of KM mouse tail‐thrombus slices which were collected and stained with FITC‐labeled free tPA, FITC‐labeled Lip@tPA, cRGD‐Lip@tPA, NE@tPA and mBiNE@tPA (equivalent tPA dose of 25 µg mL^−1^), respectively, at 37°C for 35 min. Scale bar, 100 µm. (q) MFI in the fluorescence images (l‐p), as analyzed by Fiji. (r‐t) Intensity profiles in the cross‐section regions (dotted lines) in the fluorescence images (l, o, and p), as analyzed by Fiji. Data are presented as the average ± standard deviation (n ≥ 3). Statistical analysis was performed by ANOVA test (*p < 0.05, **p < 0.01, ***p < 0.001, and ****p < 0.0001).

The mBiNE@tPA‐based “two‐birds‐one‐stone” approach combining homologous and active thrombus targeting was first shown under physiological flow conditions in a microfluidics system, which was specifically designed for thrombosis and thrombolysis research [35]. The non‐occlusive thrombi (Figure 4a) formed in the collagen‐coated biochip channels (Figure S15) at a shear rate of 1000 s^−1^ were monitored through fluorescence microscopy (Figure 4b). The tPA‐loaded liposome (denoted as Lip@tPA) and cRGD‐coated, tPA‐loaded liposome (denoted as cRGD‐Lip@tPA) that exhibited similar particle size (Figure S16a) and zeta potential (Figure S16b) to NE@tPA and mBiNE@tPA were employed for comparison. The four respective targeting modes of FITC‐labeled Lip@tPA (no thrombus targeting), cRGD‐Lip@tPA (active thrombus targeting), NE@tPA (homologous thrombus targeting) and mBiNE@tPA (bi‐specific thrombus targeting) after their perfusion in the thrombus‐containing channels, as illustrated in Figure 4c–f, were visualized by fluorescence imaging and quantified by MFI analysis (Figure 4g,h). As expected, negligible thrombus‐specific green fluorescence was detected after perfusion with Lip@tPA made from the synthetic liposomal membrane. The capability of NE@tPA for homologous thrombus targeting due to its surrounding natural erythrocyte membrane was demonstrated under physiological flow conditions, showing an 8.7‐time higher MFI than Lip@tPA and a comparable level to cRGD‐Lip@tPA. By comparison, the mBiNE@tPA‐treated thrombi showed the brightest fluorescence due to its combination of homologous and active thrombus targeting, with 1.9‐fold enhancement in thrombus accumulation as compared to the mono‐specific NE@tPA and cRGD‐Lip@tPA after perfusion under physiological flow conditions for only 5 min.

Pharmacokinetics in SD rats were evaluated by measuring the FITC‐labeled tPA concentrations in plasma after intravenous injection (Figure 4i,j). The circulation half‐lives of all tPA‐nanoformulations tested were substantially longer than free tPA, ranked in the order of: t_1/2, mBiNE@tPA_ (113.7 ± 38.1 min) ≈ t_1/2, NE@tPA_ (104.1 ± 38.5 min) > t_1/2, cRGD‐Lip@tPA_ (81.5 ± 33.8 min) ≈ t_1/2, Lip@tPA_ (77.7 ± 25.9 min) > t_1/2, free tPA_ (2.3 ± 0.8 min). The superior pharmacokinetic profiles of NE@tPA and mBiNE@tPA were attributed to the camouflaging nature of the surrounding natural erythrocyte membrane [38]. Due to the use of healthy SD rats without thrombi in this experiment, the presence of cRGD peptides on mBiNE@tPA caused no significant improvement on the pharmacokinetic profile as compared with NE@tPA. The SD rats were then sacrificed at 4 h post‐injection to track the distribution of FITC‐labeled tPA in vivo. As shown in Figure 4k, tPA mainly accumulated in the metabolic organs (i.e., kidney and liver) for the rats injected with NE@tPA and mBiNE@tPA, respectively, and demonstrated significantly higher tPA concentrations in those major organs than free tPA.

A mouse tail thrombosis model was then employed to further demonstrate the bi‐specific thrombus targeting of mBiNE@tPA. Briefly, after being starved for 8 h, KM mice were treated with fresh carrageenan via intraperitoneal injection to induce a black‐tailed thrombus. The tails with thrombi were collected, decalcified, cut into small slices, and then stained with FITC‐labeled tPA, Lip@tPA, cRGD‐Lip@tPA, NE@tPA and mBiNE@tPA, respectively, at 37°C for 35 min. As shown in Figures 4l–t and S16c,d, treatment with free tPA or Lip@tPA caused weak green fluorescence intensity only on the thrombus periphery, which was a consequence of low thrombus affinity and inability to penetrate the thrombus. The capability of NE@tPA for homologous thrombus targeting due to its surrounding natural erythrocyte membrane enabled a significant increase in fluorescence intensity on the thrombus periphery and some signal in the thrombus interior, at a comparable level with cRGD‐Lip@tPA (Figure 4q). By comparison, the mBiNE@tPA treatment led to a significantly increased green fluorescence intensity (2.0‐fold higher than NE@tPA and 6.3‐fold higher than free tPA) and efficient distribution from the thrombus periphery to the core. This result was further consolidated by using the formulations labeled with a near‐infrared fluorophore Cy5.5, which is well separated from the green autofluorescence of mouse tissues. As shown in Figure S17, the Cy5.5‐labeled mBiNE@tPA exhibited identical targeting behavior to its FITC‐labeled version, showing considerably enhanced thrombus accumulation and penetration compared to the control formulations. The excellent consistency between the two labelling approaches using spectrally distinct fluorophores confirms that the observed efficient thrombus targeting was the genuine property of mBiNE@tPA rather than a methodological artifact.

The superior thrombus accumulation and penetration of mBiNE@tPA shown in Figure 4 were attributed to the “two‐birds‐one‐stone” approach that combines erythrocyte membrane‐mediated homologous targeting and cRGD‐based active targeting. The homologous targeting capability facilitates the initial recognition and docking to the thrombus site. This process is driven not by a single receptor‐ligand pair, but by the collective, multivalent interactions conferred by the complex biochemical landscape of the erythrocyte membrane cloak. These inherent interactions allow the nanoerythrosome to engage with multiple components enriched in the thrombotic microenvironment, such as exposed cellular surfaces and secreted proteins, mimicking the natural, multifaceted involvement of erythrocytes in thrombosis. This multi‐faceted homologous targeting led to a high local concentration of mBiNE@tPA at a thrombus site. The subsequent “molecular‐level adhesion” phase enabled the specific targeting of cRGD ligands to the activated platelets. This strategy, whereby docking through the natural erythrocyte membrane allowed for efficient adhesion through engineered recognition, proved substantially more efficient than the single‐targeting approach. The synergistic interplay between complementary targeting mechanisms could enhance the thrombus penetration, representing a significant advantage over the single‐targeting approach and providing a mechanistic explanation for the effective thrombolytic activity of mBiNE@tPA.

### 3.5 Targeted Thrombolysis In Vivo

**FIGURE 5:**
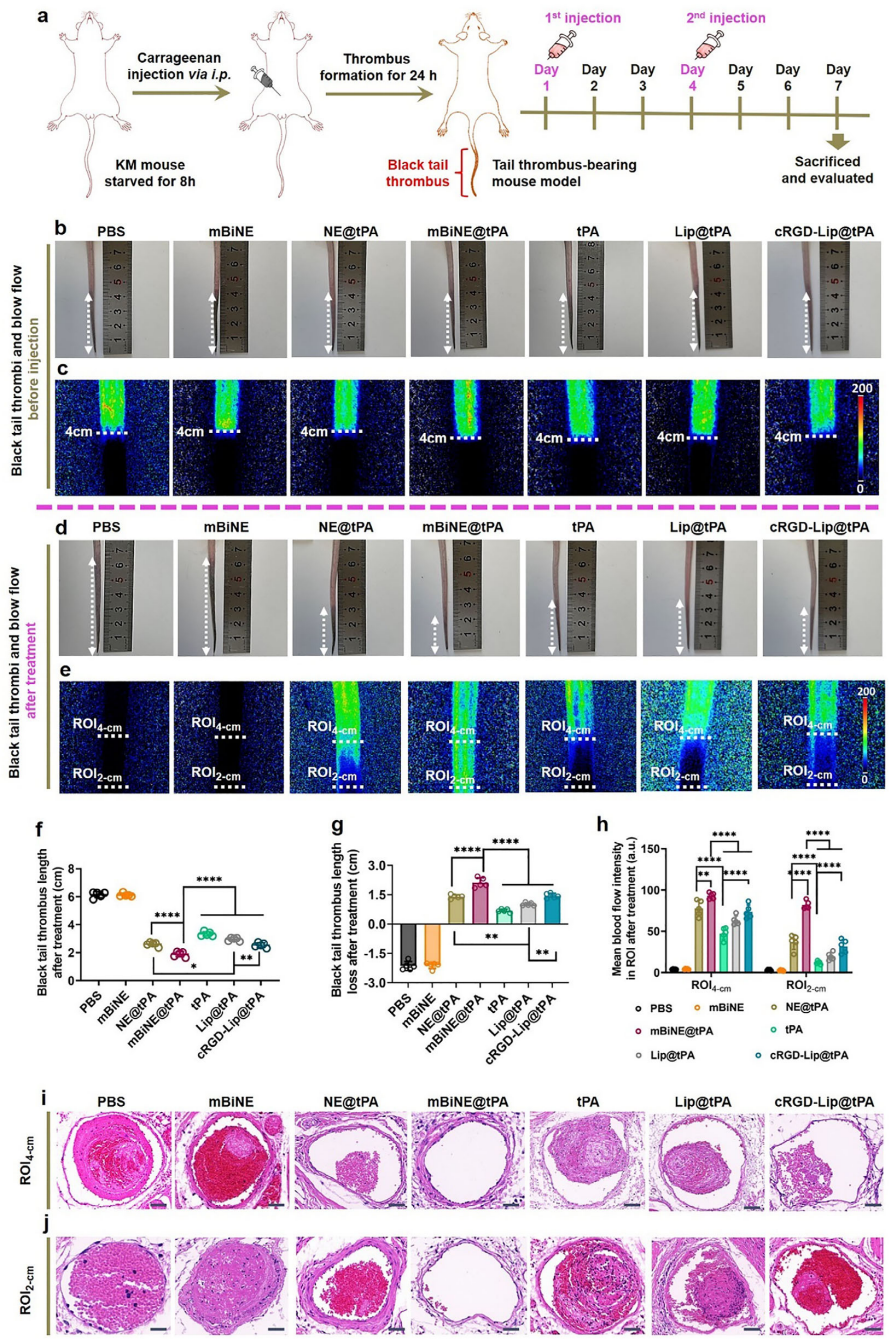
Targeted thrombolysis in the mouse tail thrombosis model. (a) Illustration of a KM mouse tail thrombosis model established via intraperitoneal (i.p.) injection with fresh carrageenan (20 mg kg^−1^). The tail thrombus‐bearing KM mice were randomly divided into 7 groups for intravenous injection with PBS buffer, mBiNE (empty vehicle control), NE@tPA, mBiNE@tPA, free tPA, Lip@tPA, and cRGD‐Lip@tPA, respectively, on days 1 and 4. The equivalent tPA dose was 10 mg kg^−1^ for each injection. (b) Black tail thrombus photographs and (c) blood flow images in 7 mouse groups before sample injection. (d) Black tail thrombus photographs, (e) blood flow images, (f) black tail thrombus lengths, (g) black tail thrombus length losses, and (h) mean blood flow intensities in the ROI_4‐cm_ and ROI_2‐cm_ in 7 mouse groups after 7 days of treatment. Representative H&E staining photomicrographs of the slices of tail thrombi in the (i) ROI_4‐cm_ and (j) ROI_2‐cm_ in 7 mouse groups after 7 days of treatment. Scale bar, 50 µm. Data are presented as the average ± standard deviation (*n* = 5). Statistical analysis was performed by the ANOVA test (*p < 0.05, ** *p* < 0.01, and ****p < 0.0001).

A tail thrombus‐bearing mouse model (Figure 5a) was utilized to evaluate the targeted thrombolytic activity of the bi‐specific mBiNE@tPA in vivo. Before injection, the initial lengths of black tail thrombi in all seven randomly divided mouse groups were controlled to be approximately 4 cm (Figures 5b and S18), and the initial mean blood flow intensity at ROI_4‐cm_, a region of interest at the 4‐cm location of mouse tail, was confirmed to be all at a negligible level (Figures 5c and S19a–S19b). The seven groups of tail thrombus‐bearing mice were treated with PBS buffer, mBiNE (empty vehicle control), NE@tPA, mBiNE@tPA, free tPA, Lip@tPA, and cRGD‐Lip@tPA, respectively, via intravenous injection on days 1 and 4. As shown in Figure 5d,f, after 7 days of treatment, the black tail thrombus length in control groups (PBS buffer and mBiNE) kept increasing from 4.00 to 6.14 ± 0.22 and 6.12 ± 0.13 cm, respectively. By comparison, the other treatment groups caused a significant reduction in the length of tail thrombi: mBiNE@tPA (1.89 ± 0.19 cm) < cRGD‐Lip@tPA (2.54 ± 0.17 cm) ≈ NE@tPA (2.61 ± 0.15 cm) < Lip@tPA (2.95 ± 0.13 cm) < free tPA (3.34 ± 0.16 cm). Among these, mBiNE@tPA exhibited the most efficient thrombolytic effect with the largest tail thrombus length loss (Figure 5g). These results were consistent with the levels of vascular recanalization at ROI_4‐cm_ and ROI_2‐cm_. As shown in Figure 5e,h, the control groups (PBS buffer and mBiNE) led to a negligible blood flow intensity at ROI_4‐cm_ and ROI_2‐cm_. The mono‐specific NE@tPA and cRGD‐Lip@tPA induced a significantly improved blood flow recovery as compared to free tPA at both tail locations tested, although their resulting mean blood flow intensities at ROI_2‐cm_ were still substantially lower than those at ROI_4‐cm_. By contrast, the significantly enhanced mean blood flow intensities after treatment with the bi‐specific mBiNE@tPA caused a similar intensity level at ROI_4‐cm_ and ROI_2‐cm_, comparable with the region without thrombi (above the 4‐cm location), demonstrating a superior blood flow recovery over the rest of the treatment groups. Figure 5i,j show the hematoxylin‐eosin (H&E) staining photomicrographs of the mouse tail slices after 7 days of treatment. It was confirmed that treatment with mBiNE@tPA resulted in complete thrombus removal at both ROI_4‐cm_ and ROI_2‐cm_. The other tPA‐formulations only caused the partial dissolution of thrombi, with a relatively smaller thrombus size at ROI_4‐cm_. In addition, the acute thrombolytic efficacy of mBiNE@tPA was assessed at earlier timepoints (1, 6, 12, 18, and 24 h). Figure S20 demonstrates rapid and significant thrombus regression, thereby validating the platform's potency in a more physiologically relevant timeframe.

The superior in vivo thrombolytic outcome of mBiNE@tPA resulted from its enhanced thrombus accumulation and penetration shown in Figure 4 via the synergistic “two‐birds‐one‐stone” targeting strategy. While mono‐specific NE@tPA and cRGD‐Lip@tPA improved the thrombus docking through erythrocyte membranes and the molecular‐level adhesion through ligand‐receptor binding separately, their effects were limited to partial, superficial thrombolysis, as evidenced by the fragmentary residual thrombi (Figure 5i,j). By contrast, bi‐specific mBiNE@tPA, which combined the thrombus docking via homologous targeting and the molecular‐level adhesion via active targeting, achieved efficient surface and interior thrombolysis. This resulted in the thorough destruction of thrombus architecture, leading to the observed complete dissolution and superior blood flow recovery.

### 3.6 Computational Simulation of Pharmacokinetics Profiles and Targeted Thrombolysis In Vivo

To elucidate deeper insights into the mechanisms responsible for the enhanced thrombolytic efficacy of mBiNE@tPA, we employed our experimentally validated computational model. The following simulations do not represent standalone predictions but were the result of a model whose parameters were derived from and validated against our experimental findings. Having calibrated and validated our computational model against in vitro experimental data, we combined a systemic PK‐PD model (Figure 1g) and a local thrombolysis model (Figure 1h) to simulate targeted thrombolysis of mBiNE@tPA in vivo. The systemic PK‐PD model applied a central compartment model to calculate the concentration profiles of mBiNE@tPA and other fibrinolysis species in the plasma phase, which were used as the inlet boundary condition for the local thrombolysis model. Figure S21 shows the flow chart of the numerical procedure employed. Details of boundary and initial conditions and other model parameters can be found in Section S2.6. The concentration profiles of free tPA and mBiNE@tPA from the systemic PK‐PD model are shown in Figures S22a and S22b, respectively. Owing to mBiNE@tPA's considerably longer half‐life, the concentration of mBiNE@tPA in plasma was sustained at a much higher level than that of free tPA. Simulation results regarding the PK‐PD profiles with free tPA and mBiNE@tPA showed good agreement with the experimental data.

The local thrombolysis model accounted for convection, diffusion, and reaction within the geometry of the mouse tail thrombus shown in Figures S23 and S24. The mouse tail thrombus was idealized to a concentric cone geometry to represent the thrombosed region between the mouse tail and coccyx. Since the real thrombus properties are unknown, the initial fibrin volume fraction was varied from 0.001 (coarse) to 0.01 (dense) to simulate thrombi with different fibrin densities, at a fixed platelet volume fraction (0.05). This was followed by simulations at two platelet volume fractions, 0.01and 0.05, at a fixed fibrin volume fraction (0.01), to represent platelet‐poor and platelet‐rich thrombi, respectively. These values were within the range found in the literature [72, 73, 74]. The inlet velocity was estimated as 0.11 m s^−1^ according to the literature value of blood flow velocity in the ventral caudal artery [75]. The diffusivity of mBiNE@tPA was assumed to be 4.11 × 10^−12^ m^2^ s^−1^, which matched values of diffusivity of other erythrocyte‐based nanoparticles reported in the literature [76]. Details of boundary and initial conditions and other model parameters can be found in Section S2. As shown in Figures S25 and S26, during the 7 days of treatment, mBiNE@tPA prevented plasma reactions of tPA and ensured higher plasminogen (PLG) and fibrinogen (FBG) levels in plasma than those of free tPA treatment, indicating a lower intracranial hemorrhage (ICH) risk during the treatment. High PLG supply can also accelerate the rate of thrombolysis.

**FIGURE 6:**
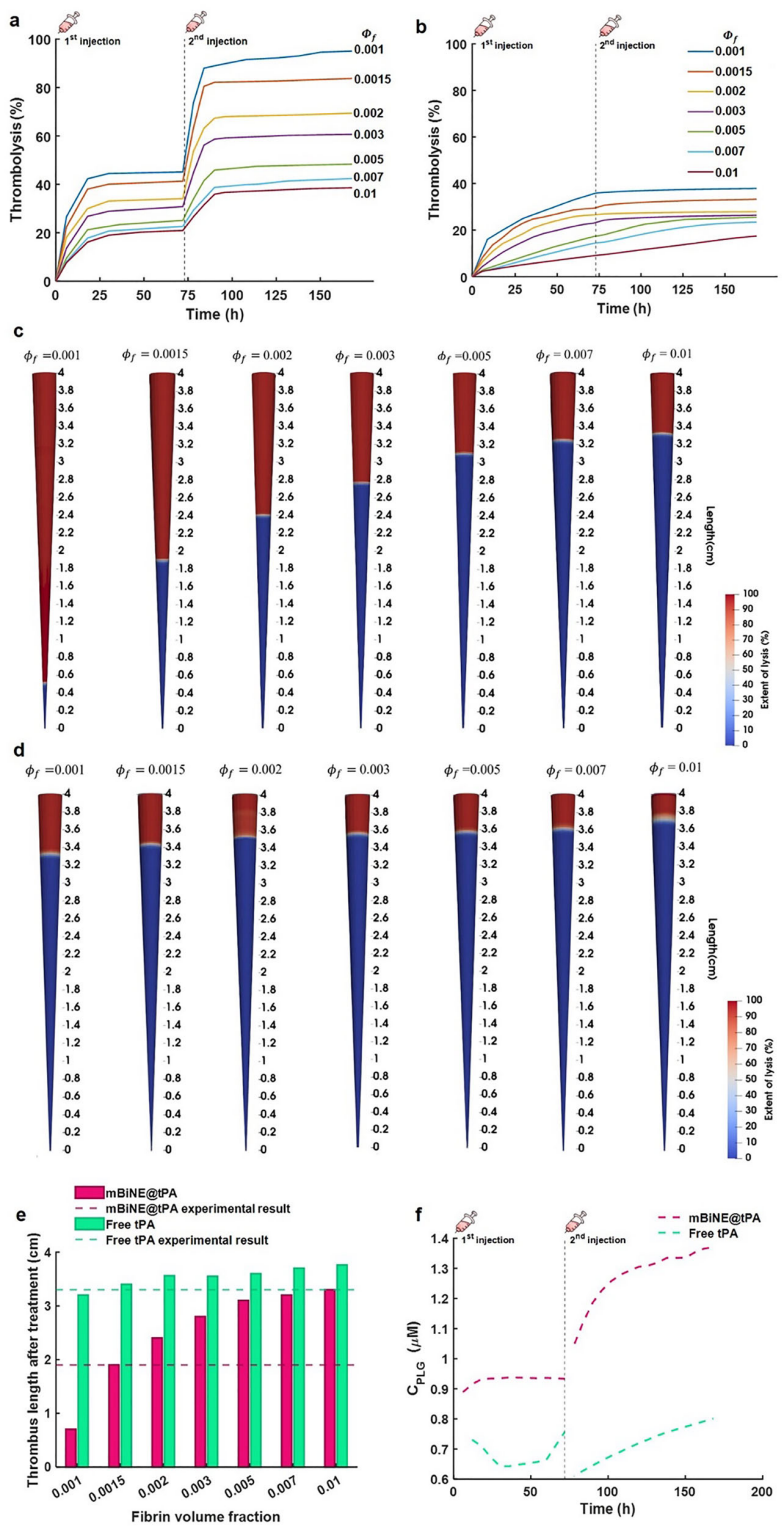
Computational simulation results of targeted thrombolysis in the mouse tail thrombosis model. Blood clot lysis by (a) mBiNE@tPA and (b) free tPA during 7 days of treatment. Remaining tail thrombus lengths after 7 days of treatment with (c) mBiNE@tPA and (d) free tPA, respectively, with the fibrin volume fraction *Φ_f_* varying from 0.001 to 0.01 at a fixed platelet volume fraction *Φ_p_* of 0.05. (e) Comparison of model predictions and experimental results of remaining tail thrombus lengths after 7 days of treatment with mBiNE@tPA and free tPA, respectively, as a function of fibrin volume fraction *Φ_f_* at a fixed platelet volume fraction *Φ_p_* of 0.05. (f) Model predictions of average PLG concentrations during 7 days of treatment with mBiNE@tPA and free tPA, respectively, at *Φ_f_* = 0.0015 and *Φ_p_* = 0.05. The simulation data were sampled every 6 h.

Figure 6 shows the simulation results for local thrombolysis in the mouse tail thrombosis model. Variations of the extent of thrombolysis over time for mBiNE@tPA and free tPA are presented in Figure 6a,b, respectively. Both mBiNE@tPA and free tPA lysed coarse thrombi faster than dense thrombi. In particular, mBiNE@tPA had a much higher lysis rate than that of free tPA in the first 20 h, and the rate decreased dramatically before the second injection, which boosted the lysis rate again in the 20 h after the second injection. On the contrary, the second injection of free tPA did not enhance the lysis as much as the first injection. The lysis rate of free tPA for dense thrombi was consistent and stable over 7 days, while the lysis rate of coarse thrombi decayed gradually with time. As shown in Figure 6c,d, the predicted remaining lengths of tail thrombi were in the range of 0.5 and 3.3 cm when the fibrin volume fraction varied from 0.001 to 0.01 after 7‐day treatment with mBiNE@tPA. By contrast, the remaining thrombus lengths after 7‐day treatment with free tPA were much larger, varying between 3.3 and 3.7 cm. Among them, as shown in Figure 6e, the remaining lengths of tail thrombi with a fibrin volume fraction of 0.0015 were closest to the experimental results (∼1.9 cm after mBiNE@tPA treatment and ∼3.4 cm after free tPA treatment). Movies S1 and S2 show temporal and spatial variations of the extent of thrombolysis during 7 days of treatment with free tPA and mBiNE@tPA, respectively.

The thrombus with a fibrin volume fraction of 0.0015 was chosen, and concentration variations of plasma phase tPA (*C_tPA_*), plasma phase PLG (*C_PLG_*), fibrin‐bound tPA (*n_tPA_*), and fibrin‐bound PLS (*n_PLS_*) during 7 days of treatment with mBiNE@tPA and free tPA were compared in Figures 6f and S27. Free tPA did not completely dissolve the thrombus in the proximal region before reaching the deeper region of the thrombus upon the first injection. The residual thrombi were completely dissolved after the second injection of free tPA. This can be explained by the overdepletion of PLG by free tPA's fast catalysis, as shown in Figure 6f. The average plasma phase PLG concentration in the thrombus treated with free tPA (green curve) was depleted much more quickly than that of the mBiNE@tPA treatment (red curve), especially in the first 36 h. Incomplete lysis further limited the supply of PLG and free tPA to the deeper region of the thrombus due to the lack of convective transport (see definition for clot dissolved region in Section S2.3). In contrast, mBiNE@tPA always dissolved the proximal region completely before reaching the deeper region of the thrombus. The triggered release of tPA limited the catalyzation of PLG but ensured sufficient PLG for reactions in the late stage of lysis (Figure 6f, red curve). Also, the convection‐enhanced transport allowed sufficient PLG concentrations to reach the thrombus for efficient lysis. Furthermore, mBiNE@tPA dissolved the thrombus with a lower platelet volume fraction (*Φ_p_* = 0.01) at a similar rate as a platelet‐rich thrombus (*Φ_p_* = 0.05) (Figure S28), implying that a slower tPA release due to a lower platelet concentration does not impair the efficacy of mBiNE@tPA.

**FIGURE 7:**
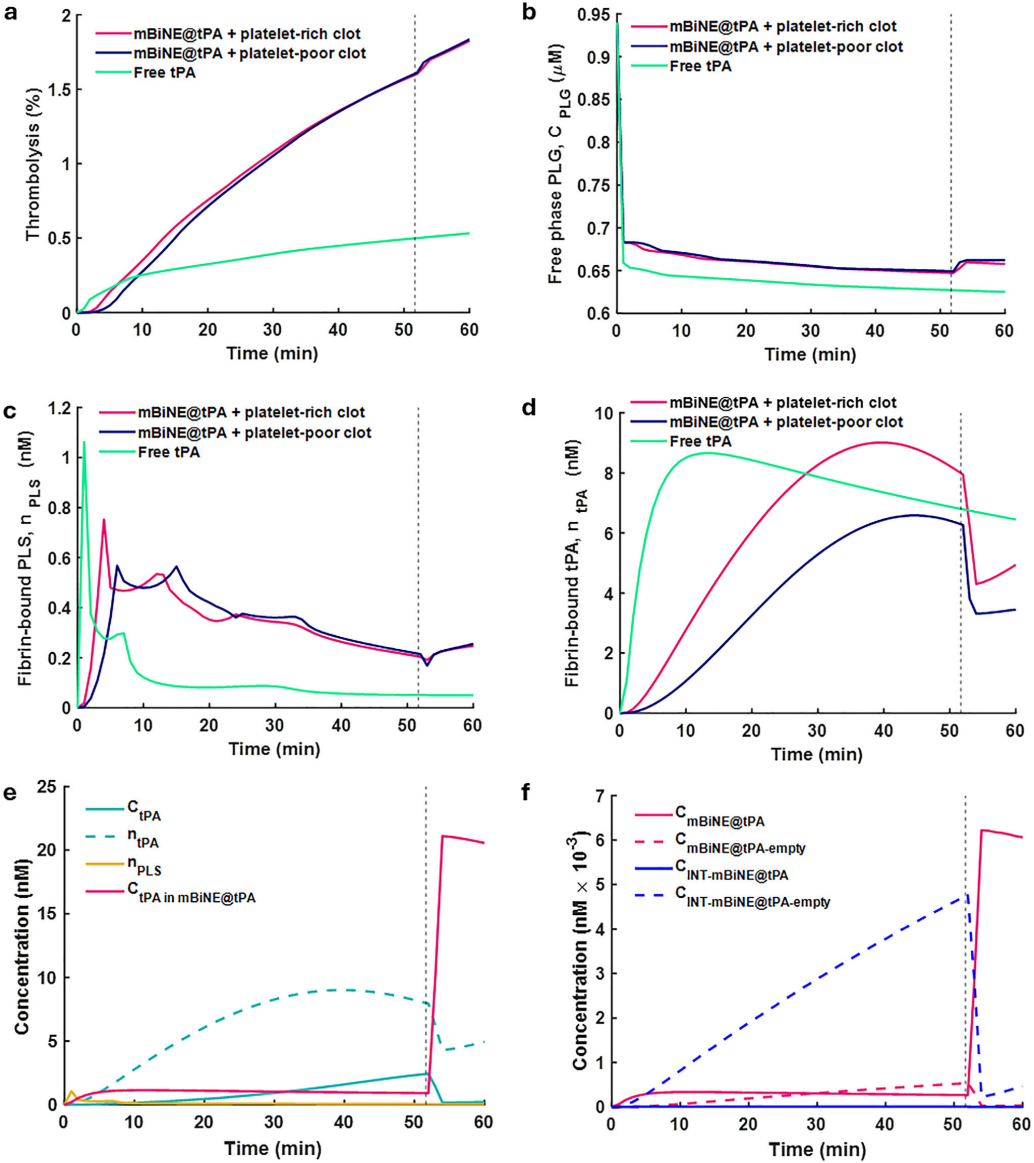
Computational simulation results of average concentrations of species during the first hour of treatment in the mouse tail thrombosis model. Real‐time changes in (a) the extent of thrombolysis, and the concentrations of (b) free PLG, (c) fibrin‐bound PLS, and (d) fibrin‐bound tPA after treatment with mBiNE@tPA and free tPA, respectively. (e) Temporal concentrations of tPA in the plasma phase (*C_tPA_*), fibrin‐bound tPA (n_tPA_), fibrin‐bound PLS (n_PLS_), and encapsulated tPA in the mBiNE@tPA (C_tPA in mBiNE@tPA_) after treatment of platelet‐rich dense thrombi with mBiNE@tPA. (f) Temporal concentrations of mBiNE@tPA (C_mBiNE@tPA_), mBiNE (C_mBiNE‐empty_), INT‐bound mBiNE@tPA (C_INT‐mBiNE@tPA_), and INT‐bound mBiNE (C_INT‐mBiNE‐empty_) in the thrombus phase after treatment of platelet‐rich dense thrombi with mBiNE@tPA. The black vertical dotted line is the time point of 3100 s. Each variable displayed in (a–f) represents its average value over the original thrombus volume. Two platelet volume fractions, 0.01 and 0.05, at a fixed fibrin volume fraction of 0.01 were chosen to represent platelet‐poor and platelet‐rich dense thrombi, respectively.

To further understand the reason behind the superior targeted thrombolytic performance of mBiNE@tPA, we investigated the temporal variations in average concentrations of key fibrinolysis proteases over the original thrombus volume and the extent of thrombolysis during the first hour post‐injection into mice with platelet‐rich or platelet‐poor dense thrombi (Figure 7a–d). With mBiNE@tPA, the front face of the thrombus was completely dissolved at the time point of 3100 s (indicated by the black vertical dotted line). Afterwards, convective transport introduced into the mouse tail domain caused a sudden increase in mBiNE@tPA concentration (Figure 7e,f). At the same time, partial recanalization of the blood vessels restored local blood circulation, which takes away the fibrinolysis species in the dissolved domain, resulting in a quick drop of the concentrations of tPA and other species after 3100 s (Figure 7d,e). Figure 7a shows that thrombolysis with free tPA was faster than with mBiNE@tPA in the first 200 s, at the expense of more PLG depletion (Figure 7b) and much higher fibrin‐bound PLS concentration (Figure 7c). However, rapid consumption and insufficient supply of PLG from the inlet resulted in a dramatic decrease in the fibrin‐bound PLS concentration after 200 s. mBiNE@tPA, with its unique triggered release and slow diffusion, yielded higher fibrin‐bound PLS and tPA concentrations at the later stage (Figure 7c,d). This helped to achieve a higher lysis rate and an earlier restoration of blood flow compared to free tPA, which further enhanced mass transfer. Interestingly, by comparing results for the platelet‐rich and ‐poor thrombi, we found that the platelet volume fraction had a very minor influence on the rate of thrombolysis (Figure 7a). However, there was more fibrin‐bound tPA in platelet‐rich thrombi. This hints that the tPA catalyzation is no longer the determining step in the fibrinolytic reactions with mBiNE@tPA. It also means that the dose can be reduced while still achieving a similar extent of lysis. Figure 7e,f compare the concentration variations of species during the tPA‐releasing process from mBiNE@tPA in platelet‐rich thrombi. Benefiting from the high platelet affinity, most of the mBiNE@tPA quickly bound with integrin (INT) and released tPA, resulting in the accumulation of empty mBiNE on the platelets (Figure 7f, red dotted curve). Most of the tPA released from mBiNE@tPA stayed in the bound phase (Figure 7e, green dotted curve), although the bound plasmin concentration did not increase with the accumulation of fibrin‐bound tPA (Figure 7e). This further indicates that PLG deficiency hindered the lysis process. Therefore, the integration of experimental and computational approaches provided complementary insights into the performance of mBiNE@tPA. While experimental results conclusively demonstrated its superior thrombolytic efficacy, computational simulations elucidated the underlying mechanism. The agreement between model predictions and empirical data validated our proposed framework of targeting and triggered release, providing a mechanistic explanation for the more uniform clot dissolution observed experimentally.

### 3.7 In Vivo Biocompatibility, Coagulation, and Hemorrhagic Risks

**FIGURE 8:**
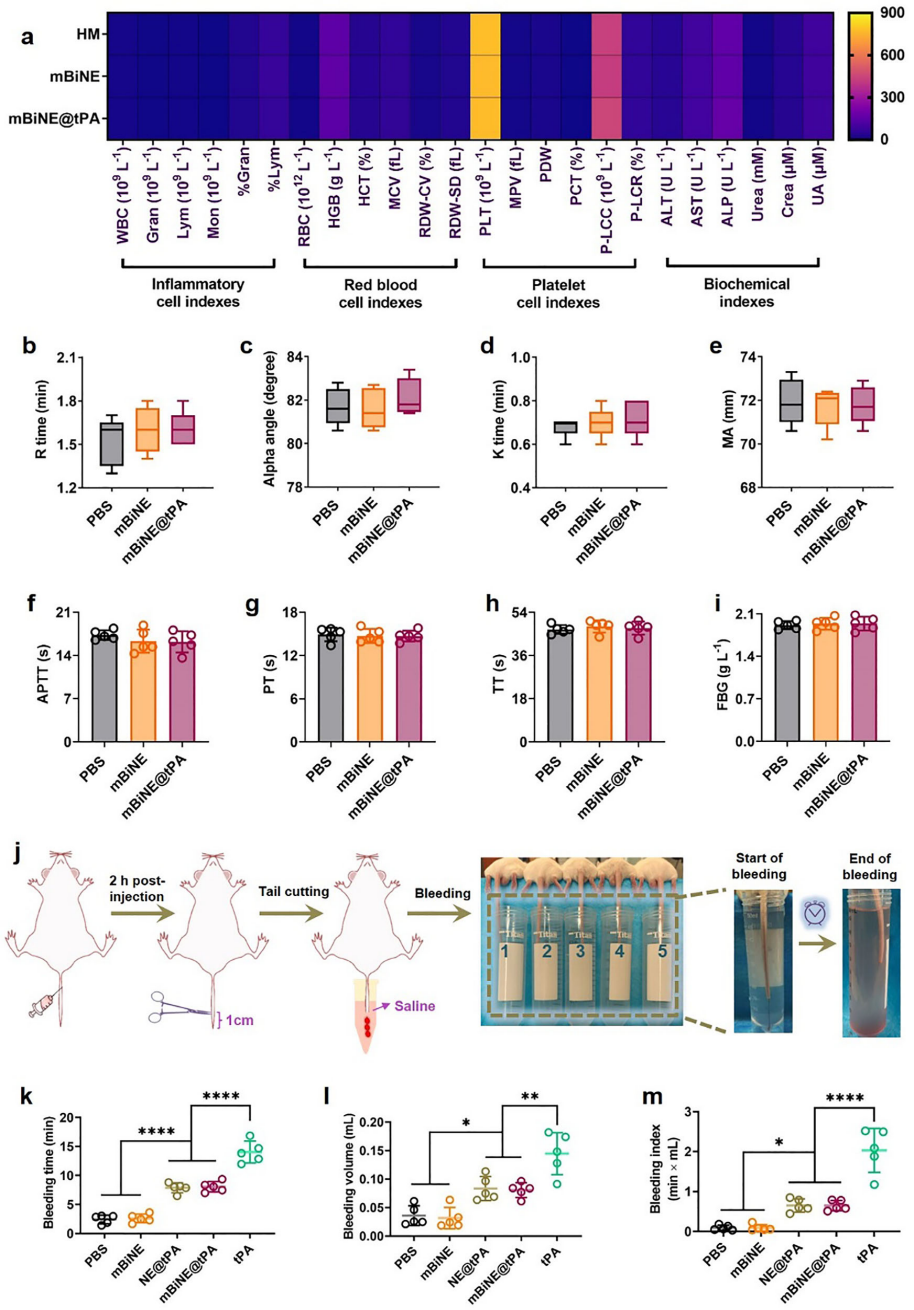
Coagulation and hemorrhagic risks in vivo. (a) Hematological indexes of white blood cells (WBC), granulocytes (Gran), lymphocytes (Lym), monocytes (Mon), granulocyte percentage (%Gran), lymphocyte percentage (%Lym), red blood cell (RBC), hemoglobin (HGB), hematocrit value (HCT), mean corpuscular volume (MCV), coefficient of variation of red blood cell volume distribution width (RDW‐CV), standard deviation of red blood cell volume distribution width (RDW‐SD), platelet (PLT), mean platelet volume (MPV), platelet distribution width (PDW), thrombocytocrit (PCT), platelet large cell count (P‐LCC) and platelet‐to‐large‐cell ratio (P‐LCR) in the blood of KM mice after 7 days of treatment with NE@tPA and mBiNE@tPA (equivalent tPA dose of 10 mg kg^−1^), respectively; Biochemical indexes of alanine aminotransferase (ALT), aspartate aminotransferase (AST), alkaline phosphatase (ALP), Urea, creatinine (Crea) and uric acid (UA) in the serum of KM mice after 7 days of treatment with NE@tPA and mBiNE@tPA (equivalent tPA dose of 10 mg kg^−1^), respectively. Healthy mice (HM) with an injection of PBS buffer under the same treatment conditions were used as controls. Data are presented as the average ± standard deviation (*n* = 5). Coagulation parameters including (b) R time, time to formation of initial fibrin threads, (c) Alpha angle, rapidity with which a thrombus forms, (d) K time, time until a thrombus reaches a strength of 20 mm, and (e) maximum amplitude (MA), maximum strength of a thrombus. Plasma coagulation indicators including (f) APTT, activated partial thromboplastin time, (g) PT, prothrombin time, (h) TT, thrombin time, and (i) FBG, fibrinogen. Intravenous injection of healthy SD rats with PBS, NE@tPA, and mBiNE@tPA, respectively, for (b–i) was at an equivalent tPA dose of 10 mg kg^−1^, followed by measurements by TEG assay and coagulometry at 24 h post‐injection. (j) Illustration of a tail bleeding assay using KM mice after amputation of the distal 1‐cm tail section, immediately followed by submergence into a 37°C saline solution. (k) Bleeding time, (l) bleeding volume, and (m) bleeding index of the tail‐transected KM mice at 2 h post intravenous injection with PBS buffer, mBiNE, NE@tPA, mBiNE@tPA, and free tPA (equivalent tPA dose of 10 mg kg^−1^), respectively. The bleeding index was defined by the product of bleeding time (min) and bleeding volume (mL). Data are presented as the average ± standard deviation (n ≥ 3). Statistical analysis was performed by the ANOVA test (*p < 0.05, ** *p* < 0.01, and ****p < 0.0001).

Mouse body weights were monitored, blood samples were collected from the abdominal aorta for hematology and biochemical tests, and the main organs were dissected for H&E staining to evaluate the biocompatibility of mBiNE@tPA in vivo. As shown in Figure S29, no noticeable body weight loss was observed in the seven mouse groups during 7 days of treatment. In the hematology study, the levels of hematological indices of the healthy KM mice in the PBS buffer treatment group (HM) were used as a control. Figure 8a shows that 7 days of treatment with mBiNE@tPA or mBiNE caused negligible changes to inflammatory cell indexes (white blood cells, WBC; granulocytes, Gran; lymphocytes, Lym; monocytes, Mon; granulocyte percentage, %Gran; and lymphocyte percentage, %Lym), red blood cell indexes (red blood cell, RBC; hemoglobin, HGB; hematocrit value, HCT; mean corpuscular volume, MCV; coefficient of variation of red blood cell volume distribution width, RDW‐CV; and standard deviation of red blood cell volume distribution width, RDW‐SD), and platelet cell indexes (platelet, PLT; mean platelet volume, MPV; platelet distribution width, PDW; thrombocytocrit, PCT; and platelet large cell count, P‐LCC; and platelet‐to‐large‐cell ratio, P‐LCR). Given the accumulation of mBiNE@tPA and NE@tPA mainly in the liver and kidney after intravenous injection, main biochemical indexes related to the liver (alanine aminotransferase, ALT; aspartate aminotransferase, AST; and alkaline phosphatase, ALP) and kidney (Urea; creatinine, Crea; and uric acid, UA) were measured, and no obvious changes were observed after 7 days of treatment. Furthermore, the H&E staining photomicrographs (Figure S30) showed that mBiNE@tPA and NE@tPA caused no obvious pathological toxicity or inflammatory cells in major organs (heart, liver, spleen, lung, and kidney). These results present mBiNE@tPA as a biologically safe platform for use in vivo.

The effect of mBiNE@tPA on coagulation was assessed by thromboelastography (TEG) assay. The healthy SD rats were intravenously injected with PBS, mBiNE, and mBiNE@tPA, respectively. At 24 h post‐injection, SD rats were anesthetized, and whole blood was collected for the TEG test. As displayed in Figure 8b–e, no significant variations in the parameters of R time, Alpha angle, K time, or maximum amplitude (MA) were observed for mBiNE@tPA and two control groups (mBiNE and PBS buffer). mBiNE@tPA was also found to cause no significant changes to plasma coagulation indicators, including activated partial thromboplastin time (APTT), prothrombin time (PT), thrombin time (TT), and fibrinogen (FBG), after 24 h of treatment as compared with the PBS buffer treatment in both SD rats (Figure 8f–i) and KM mice (Figure S31).

Furthermore, the hemorrhagic risk of mBiNE@tPA was evaluated by the tail bleeding assay [77]. As shown in Figure 8j, a distal 1‐cm segment of the KM mouse tail was amputated to cause bleeding at 2 h post intravenous injection with PBS buffer, mBiNE, NE@tPA, mBiNE@tPA, and free tPA, respectively, and the bleeding time (*t_b_*) and bleeding volume (*v_b_*) were recorded. As shown in Figure S32, at the clinically relevant tPA dose (0.9 mg kg^−1^), treatment with mBiNE@tPA resulted in bleeding parameters (i.e., bleeding time, volume, and index) statistically indistinguishable from those of the PBS control group. In stark contrast, administration of an equivalent dose of free tPA induced a significant and prolonged hemorrhagic tendency. This finding definitively shows our system's safety within the therapeutic window. Furthermore, we used a high dose (10 mg kg^−1^) to stress‐test the system. As shown in Figure 8k,l, the mice treated with the control groups, PBS buffer (*t_b, PBS_* = 2.4 ± 0.7 min, *v_b, PBS_* = 0.036 ± 0.017 mL) and mBiNE (*t_b, mBiNE_* = 2.6 ± 0.7 min, *v_b, mBiNE_* = 0.032 ± 0.019 mL), showed rapid complete blood coagulation due to intrinsic hemostatic functions (Figure 8k,l). Treatment with free tPA caused the longest bleeding time (*t_b, tPA_* = 14.0 ± 1.9 min) and highest bleeding volume (*v_b, tPA_* = 0.145 ± 0.0367 mL). The bleeding time and volume after treatment with NE@tPA (*t_b, NE@tPA_* = 7.9 ± 0.9 min, *v_b, NE@tPA_* = 0.083 ± 0.021 mL) or mBiNE@tPA (*t_b, mBiNE@tPA_* = 8.1 ± 0.9 min, *v_b, mBiNE@tPA_* = 0.081 ± 0.013 mL) were significantly decreased as compared to free tPA. A bleeding index, which is the product of bleeding time and bleeding volume, was calculated to quantify the hemorrhagic risk [78]. As seen in Figure 8m, the bleeding index in the mice treated with mBiNE@tPA was only 0.6 ± 0.1 min × mL, which was 3.5‐fold lower than that of the free tPA treatment group (2.1 ± 0.5 min × mL). Thus, it can be seen that, even under the extreme condition, mBiNE@tPA showed a markedly improved safety margin over free tPA. Therefore, the combined data robustly confirm that our “two‐birds‐one‐stone” strategy not only enhances efficacy but, more importantly, achieves a therapeutically viable safety profile. It is worth noting that our serum stability data shown in Figure 2g demonstrated the excellent integrity of mBiNE@tPA with no significant tPA leakage over 24 h in serum at 37°C under shaking at 120 rpm. Hence, the markedly reduced hemorrhagic risk of mBiNE@tPA as compared to free tPA was due to the controlled, site‐specific tPA release rather than non‐specific leakage during circulation. This targeted release profile of mBiNE@tPA enabled high tPA concentrations at a thrombus site while minimizing systemic exposure, representing a crucial safety advantage over conventional tPA therapy. This suggests that the bio‐inspired nanomedicine mBiNE@tPA can remarkably reduce the hemorrhagic side‐effect of free tPA, thereby enabling targeted thrombolytic therapy for safer and more effective treatment of catastrophic events including stroke, myocardial infarction and pulmonary embolism. Compared to molecularly targeted approaches (e.g., single‐chain antibody‐urokinase fusions or nanoparticles) [79, 80], our mBiNE@tPA platform offers a complementary, biomimetic alternative. It is important to note that our system uniquely integrates a scalable membrane‐based fabrication, the inherent long circulation of erythrocyte membranes, and a dual‐targeting mechanism (homologous and active thrombus targeting) for enhanced thrombus accumulation in vivo. Thus, mBiNE@tPA represents a valuable expansion of the targeted thrombolysis toolkit.

## 4 Conclusion and Future Perspectives

In summary, a “two‐birds‐one‐stone” approach that combines homologous and active thrombus targeting for effective targeted thrombolysis was first developed. A tPA‐loaded multivalent bi‐specific nanoerythrosome, denoted as mBiNE@tPA, was successfully fabricated by a simple and scalable method to exploit two main thrombus components by utilizing the natural erythrocyte membrane for homologous targeting and fibrinogen‐derived cRGD motifs for active targeting. This bio‐inspired mBiNE@tPA was small with a narrow size distribution, negative surface charge, and good stability. Flow cytometry, CLSM, microfluidic system under physiological flow conditions, and ex vivo thrombus fluorescence imaging showed efficient thrombus targeting and enhanced thrombus penetration of mBiNE@tPA by combining inherent homologous thrombus targeting due to the presence of erythrocyte components and active thrombus targeting due to cRGD binding to GPIIb‐IIIa on activated platelets at a thrombus site. Efficient tPA release was shown to be triggered by activated platelets through membrane fusion, and the pharmacokinetic profiles displayed that mBiNE@tPA significantly prolonged circulation time compared to free tPA. Meanwhile, the activated platelet‐triggered tPA release and improved pharmacokinetic profile of mBiNE@tPA were reproduced by a purpose‐built computational model. Effective in vitro targeted fibrinolytic and thrombolytic activities were demonstrated in the fibrin clot and halo blood clot models. It was demonstrated that mBiNE@tPA significantly enhanced thrombolytic therapy as compared to single or no targeting specificity and free tPA, and finally enabled complete vascular recanalization in a well‐established mouse model, which was also elucidated elaborately by our computational modeling. Besides, intravenous administration of mBiNE@tPA displayed low coagulation and hemorrhagic risks, and excellent biocompatibility in vivo.

However, the present study of this “two‐birds‐one‐stone” system still faces some limitations. Firstly, it is important to acknowledge that while upregulation of the GPIIb/IIIa (α_IIb_β_3_) integrin is a hallmark of activated platelets at a thrombus site [81, 82], and cRGD has demonstrated a higher specificity for α_IIb_β_3_ than its linear analogues [83], it is not an absolutely specific ligand. Its promiscuity for other integrins (e.g., α_v_β3, α_5_β_1_) is a recognized limitation of the current design. Future work could explore ligands with a higher specificity, such as the single‐chain antibody against activated GPIIb/IIIa [79], to potentially further enhance thrombus targeting precision. Our rationale for selecting cRGD for the nanoerythrosome coating was grounded in its proven efficacy in thrombus targeting, ease of conjugation, and potential for a broader affinity to enhance its overall avidity within the complex thrombus microenvironment [60, 61, 62, 63, 84, 85]. The superior thrombolytic efficacy and safety profile of mBiNE@tPA in vivo, shown in the present work, have validated our strategy, yet the pursuit of even more specific targeting ligands remains a promising avenue to future optimization of this platform. Secondly, while the carrageenan‐induced tail thrombosis model provided a reproducible in vivo system for the initial validation of our targeting strategy and comparative efficacy, it is important to acknowledge its limitations in fully recapitulating the complexity of human pathological thrombi. This model primarily generates fibrin‐ and platelet‐rich clots, which underrepresent the significant erythrocyte‐rich components prevalent in many clinical scenarios, particularly venous thromboembolism [86, 87]. Consequently, the present in vivo data may not fully capture the performance of mBiNE@tPA in such RBC‐dense environments, for which the homologous targeting mechanism might offer even greater advantages.

To bridge the translational gap, future work will prioritize the evaluation of mBiNE@tPA in more physiologically relevant in vitro thrombosis models (e.g. endothelialized microfluidic model) [88], and more clinically relevant animal models, such as the ischemic stroke animal model by photothrombosis [89] or FeCl_3_‐induced arterial injury model [88, 90], which are known to produce heterogenous, mixed‐composition thrombi [91]. Furthermore, the robustness of our platform across a spectrum of thrombotic environments was supported by our computational simulations. As displayed in Figures 6 and 7, our computational models explicitly accounted for variations in thrombus composition, including fibrin and platelet densities. The simulations consistently showed the superior thrombolytic efficacy of mBiNE@tPA over a range of parameters, suggesting its potential utility is not limited to a single thrombus phenotype. This in silico evidence underscores the promise of our “two‐birds‐one‐stone” strategy for targeting the diverse thrombi encountered in human disease and provides a compelling rationale for its continued development. Collectively, this multivalent bi‐specific nanoerythrosome represents a promising strategy for targeted therapies.

## Author Contributions

R.C., X.Y.X., S.A.T., and Y.H. conceived the project. R.C. guided and supervised the project. X.Y.X., Y.L., and Y.H. co‐supervised the project. R.C., Y.H., N.H., and Y.L. designed the experiments. X.Y.X., Y.Y., and B.G. designed the computational modeling. Y.H. carried out the synthesis and characterization of nanoerythrosomes, investigated their selective binding, triggered tPA release, and targeted thrombolysis both in vitro and in vivo. Y.Y. performed the computational simulations. Y.H., R.C., and Y.L. designed and carried out the animal study. N.H. carried out scalable production using a benchtop pressure‐driven extruder designed in‐house, SDS‐PAGE, and cryo‐TEM studies. J.W., Y.G., and C.L. assisted with the halo blood clot model and fibrinolysis analysis in vitro. J.W. and Y.G. assisted with the coagulation and hemorrhagic risk studies. R.C., Y.H., Y.Y., N.H., B.G., Y.L, and X.Y.X. analyzed the data and wrote the manuscript. All authors discussed the results and edited the manuscript.

## Conflicts of Interest

R.C., Y.H., X.Y.X., and S.A.T. are listed as joint inventors of the patent (European Application Number: 20838205, and US Application Number: 17/7864031) related to the red blood cell‐derived vesicle for targeted treatment of thrombotic disorders. The authors declare no other conflicts of interest.

## Supporting information




**Supporting File 1**: smll74578‐sup‐0001‐SuppMat.docx.

**Supporting File 2**: smll74578‐sup‐0002‐MovieS1.mp4.

**Supporting File 3**: smll74578‐sup‐0003‐MovieS2.mp4.

## Data Availability

The data that support the findings of this study are available from the corresponding author upon reasonable request.
