# Supplementary material for: Multivalent Bi‐Specific Nanoerythrosomes: A Two‐Birds‐One‐Stone Approach to Combine Homologous and Active Targeting for Effective Thrombolysis
Source: Small. 2026 Jul 13;22(47):e74578. doi: 10.1002/smll.74578 (PMC13495915; doi:10.1002/smll.74578)
Supplement: Supplementary file 1 [file SMLL-22-e74578-s003.docx]

Supporting Information

Multivalent bi-specific nanoerythrosomes: a two-birds-one-stone approach to combine homologous and active targeting for effective thrombolysis

Yu Huang^1,2,†,^*, Yilin Yang^1,†^, Nicole Henry^1,†^, Boram Gu^3^, Jiahua Wang^2^, Yuanyuan Guo^2^, Colin Longstaff^4^, Simon A. Thom^5^, Yuehua Li^2,^*, Xiao Yun Xu^1,^* and Rongjun Chen^1,^*

^1^ Department of Chemical Engineering, Imperial College London, South Kensington Campus, London SW7 2AZ, United Kingdom

^2^ Department of Radiology, Shanghai Jiao Tong University School of Medicine Affiliated Shanghai Sixth People’s Hospital, 600 Yi Shan Road, Shanghai 200233, P. R. China

^3^ School of Chemical Engineering, Chonnam National University, Gwangju 61186, Republic of Korea

^4^ Biotherapeutics Section, National Institute for Biological Standards and Control, South Mimms, Herts EN6 3QG, United Kingdom

^5^ National Heart and Lung Institute, Imperial College London, London W2 1LA, United Kingdom

* Corresponding authors: E-mail: rongjun.chen@imperial.ac.uk (R.C.); yun.xu@imperial.ac.uk (X.Y.X.); liyuehua77@sjtu.edu.cn (Y.L.); yuhuang6y@163.com (Y.H.)

^†^ These authors contributed equally to this manuscript.

**1. Supplementary experimental methods**

**1.1 Isolation of platelets**

Whole blood collected in ACD was centrifuged at 200 × g for 15 min to obtain platelet-rich plasma (PRP). 2 μM prostaglandin E1 was added to the PRP to inhibit platelet aggregation and platelets were then collected by further centrifugation 1000 × g for 15 min. The isolated platelets were re-suspended in PBS and counted.

**1.2** **Synthesis of FITC-labeled tPA**

FITC-labeled tPA was synthesized by the reaction of the isothiocyanate group (-N=C=S) on FITC with primary amine groups (-NH_2_) on tPA in alkaline buffer (pH 8.0) to form a stable thiourea bond. Briefly, 1 mL of FITC solution (1.0 mg mL^-1^) in DMSO was added dropwise into 2 mL of tPA solution (1.0 mg mL^-1^) in PBS buffer at pH 8.0 and stirred overnight at 4 ^o^C in the dark. The resulting mixture was then extensively dialyzed (MWCO = 10 kDa) against PBS (pH 7.4) for 24 h to remove any unreacted free FITC, ensuring that the final product contained only covalently labeled FITC-tPA. The purified product was finally lyophilized.

**1.3 Trypsin protection assay**

100 µL of purified mBiNE@tPA suspension was mixed with 10 µL of trypsin solution (0.25% w/v in PBS, pH 7.4) and incubated at 37 ^o^C for 30 min under gentle shaking. Trypsin activity was then terminated by the addition of 10 µL of trypsin inhibitor (1 mg mL^-1^). After trypsin inhibition, the mBiNE@tPA samples were centrifuged at 20,000 × g for 30 min at 4 ^o^C to separate nanoerythrosomes from any potentially released fragments. The pellet was resuspended in 0.1% (v/v) Triton X-100 and incubated at 37 ^o^C for 30 min to facilitate release of the encapsulated tPA. The tPA activity was measured using the S-2251 chromogenic substrate assay. A mixture of free tPA with trypsion (positive congtrol) and a mixture of mBiNE@tPA with PBS alone (negative control), both at an equivalent concentration, were used as the positive and negative controls, respectively.

**1.4** **Stability in serum**

The stability of nanoerythrosomes in serum under dynamic situation was investigated using the previously published protocol with some modifications. ^[1,2]^ Briefly, fetal bovine serum (FBS) was placed in a water bath at 37 ^o^C under 120 rpm to simulate physiological media. The nanoerythrosomes, mBiNE@tPA and NE@tPA (0.2 mg mL^-1^), were suspended in PBS at pH 7.4, to which FBS was then added to yield the final serum concentration of 50% (v/v). The resulting mixture was incubated in a water bath at 37 ^o^C under 120 rpm for 24 h. The particle size and zeta potential of the nanoerythrosomes were measured by dynmic light scattering (DLS) using a Zetasizer Nano S (Malvern, UK) and by zeta potential analysis using a Zetasizer Lab (Malvern, UK), respectively, whearus drug leakage was evaluated using FITC‑labeled tPA, at predetermined time points (0, 3, 6, 9, 12, and 24 h).

**1.5 *In vitro* tPA leakage**

FITC‑labeled tPA was encapsulated into mBiNE@tPA and control formulations. tPA leakage measurements were performed in pH 7.4 PBS or in 50% (v/v) FBS prepared in pH 7.4 PBS at 37 °C under gentle agitation (120 rpm). At predetermined time points, an aliquot of each sample was transferred to a 100 kDa MWCO ultrafiltration tube and centrifuged at 14,000 × g for 30 min at 4 °C to separate released FITC‑tPA in the filtrate from the retained nanoerythrosomes. The fluorescence intensity of the filtrate was measured using a microplate reader (λ_ex_ = 488 nm, λ_em_ = 525 nm) (Tecan, Switzerland), and the concentration of released tPA was calculated from a standard calibration curve generated from known FITC‑tPA concentrations. The tPA leakage (%) was calculated according to the following equation:

$tPA leakage (\%)= \frac{A_{t}}{A_{i}}\times100$	(S1)

where *A*_t_ is the tPA amount released at time t; *A*_i_ is the initial loaded tPA amount.

**1.6** **Hemocompatibility assay**

Solution with varying concentrations of mBiNE@tPA were prepared in PBS at pH 7.4. Mouse blood was collected in EDTA and centrifuged at 2000 × g for 15 min to collect the erythrocytes. The isolated erythrocytes were washed three times with PBS and resuspended in the various mBiNE@tPA solutions. Negative and positive controls were prepared by resuspending erythrocytes in only PBS or deionized water, respectively. All samples were incubated in a water bath at 37 °C under 120 rpm for 1 h. Afterwards, the samples were centrifuged at 4,000 rpm (2,200 ×g) for 4 min and their supernatants were collected. The absorbance of the supernatant was measured by a plate reader at 540 nm (BioTek Epoch, USA). The hemolysis percentages were calculated by the following equation:

$Hemolysis (\%)=\frac{A_{s}{-A}_{n}}{A_{p}{-A}_{n}}\times100$	(S2)

where *A_s_* is the absorbance after incubation with samples; *A_n_* is the absorbance of negative control; *A_p_* is the absorbance of positive control.

**1.7** **MTT assay**

Human umbilical vascular endothelial cells (HUVEC) were used in an MTT assay to determine the cytotoxicity of mBiNE@tPA. HUVECs were cultured in M199 medium (Hyclone, USA) supplemented with 10% (v/v) fetal bovine serum, 100 U mL^-1^ penicillin, 100 U mL^-1^ streptomycin and 2 mM glutamine. HUVEC cells were seeded (8 × 10^3^ cells per well) in 96-well plates and incubated overnight. The cells were treated with 200 μL of fresh medium with 50 μL of serial concentrations of mBiNE@tPA overnight. 20 μL MTT assays stock solution (5 mg mL^-1^) in PBS was added to each well and left to incubate for 4 h. Next, the supernatant was removed from each well and 200 μL DMSO was added to dissolve the formazan crystals. The resulting absorbance was measured by a plate reader at 570 nm (BioTek Epoch, USA). The relative cell viability (%) was calculated using the following equation:

$Cell viability (\%)=\frac{A_{s}{-A}_{b}}{A_{c}{-A}_{b}}\times100$	(S3)

where *A*_s_ is the absorbance of the sample well (HUVEC incubated with nanoerythrosomes); *A*_b_ is the background absorbance (wells with medium only); A_c_ is the absorbance of the control well (HUVEC incubated with medium only).

**1.8 D****etermination of binding affinity with activated platelets by flow cytometry**

2 mL sheep platelets (1.0 × 10^8^ mL^-1^) were seeded in 6-well plates and incubated with 100 μL thrombin (1 U mL^-1^) for 20 min to allow for platelet activation. Nanoerythrosomes were fluorescently labeled with FITC-conjugated lipids for tracking. Inactivated platelets or thrombin-activated platelets were incubated with the FITC-labeled tPA or FITC-labeled tPA-loaded nanoerythrosomes overnight (equivalent tPA concentration of 0.2 mg mL^-1^). Free tPA or nanoerythrosomes, which were not attached to platelets, were removed by centrifugation. Data for 1.0 × 10^4^ gated events were collected and analysed using a BD Fortessa II flow cytometer. In the quantitative analysis, all fluorescence intensities were normalized relative to the corresponding PBS control, thereby effectively accounting for, and subtracting the non-specific background.

**1.9 Determination of binding affinity with activated platelets by CLSM**

2 mL sheep platelets (1.0 × 10^8^ mL^-1^) were seeded in 6-well plates, with a collagen-coated glass coverslip on the bottom of each well. After 30 min, 100 μL thrombin (1 U mL^-1^) was incubated with some of the coverslips for 20 min to ensure platelet activation. Nanoerythrosomes were fluorescently labeled with FITC-conjugated lipids for tracking. Inactivated platelets or thrombin-activated platelets were incubated with various FITC-labeled tPA-loaded nanoerythrosomes overnight (equivalent tPA concentration of 0.2 mg mL^-1^). The platelets were then fixed with a 4.0% formaldehyde solution for 30 min. The resulting coverslips were rinsed three time with PBS at pH 7.4 and mounted onto slides before imaging them with a Leica SP5 MP confocal microscope.

**1.10 GPIIb-IIIa assay**

Levels of the platelet membrane glycoprotein (GPIIb-IIIa) for platelet samples treated with various formulations: PBS, NE@tPA, mBiNE@tPA (equivalent tPA concentration of 0.2 mg mL^-1^) were determined using a GPIIb-IIIa enzyme-linked immunosorbent assay (ELISA) kit (Bohu Biological Technology, China) and its associated procedure. Briefly, 2 mL of whole blood collected from healthy SD rats was centrifuged at 200 × g for 15 min to collect PRP. 600 μL PRP was then added to 5400 μL of the diluent (from the kit). Then, 860 μL of this mixture was placed in the tube and activated by 100 μL thrombin (1 U mL^-1^) at 37 °C for 20 min. Afterwards, 40 μL of a specific formulation was then added and incubated at 37 °C for 10 min. The samples were treated according to the procedure of the GPIIb-IIIa ELISA kit, and the resulting absorbance was measured by a plate reader (BioTek Epoch, USA) at 450 nm. The level of GPIIb-IIIb for each sample was calculated by using a standard curve, which was obtained using the standard wells of the ELISA plates.

**1.****11 Activated platelet-triggered drug release**

Drug release was evaluated in the presence of activated platelets to confirm the tPA release specifically upon interaction with activated platelets. 200 μL of sheep platelets (1.0 × 10^8^ mL^-1^) were placed into a collagen-coated 96-well microplate and incubated with 20 μL thrombin (1 U mL^-1^) at 37 °C for 20 min to allow for platelet activation. The platelet activation was confirmed by the platelet aggregation assay using a Sunrise 96-well plate reader (Tecan, Switzerland) at 595 nm. Inactivated platelets or thrombin-activated platelets were incubated with various tPA-loaded nanoerythrosomes (equivalent tPA dose of 0.5 mg mL^-1^). The amount of released tPA in each well was determined by measuring the absorbance with a GloMax-Multi Microplate Multimode Reader (Promega, USA) at 405 nm, according to a chromogenic substrate S-2251 assay. The percentage of tPA release was calculated according to the following equation:

$tPA release (\%)= \frac{A- A_{c}}{A_{p}- A_{c}}\times100$	(S4)

where *A* is the absorbance of platelet samples after incubation with the tPA-loaded nanoerythrosomes; *A_c_* is the absorbance of negative control after incubation with the pH 7.4 PBS buffer only; *A_p_* is the absorbance of positive control after incubation with the tPA-loaded nanoerythrosomes lysed by Triton X-100.

**1.12 Drug release mechanism**

A fluorescence resonance energy transfer (FRET) assay was employed to confirm the membrane fusion upon interactions with activated platelets. Briefly, NBD-PE (donor) and Rhod-PE (acceptor) were coated onto the nanoerythrosome surface, each at 1 mol%, according to the mentioned method of nanoerythrosome preparation method. Then, 200 μL of sheep resting or activated platelets (1.0 × 10^8^ mL^-1^) were added to 1.8 mL of nanoerythrosome formulations under stirring. Successful fusion between the nanoerythrosome membrane and the platelet membrane will lead to a decrease in the density of the donor-acceptor pair resulting in a reduced FRET effect and consequently increasing the fluorescence intensity of the donor, NBD. The NBD fluorescence (λex = 465 nm and λem = 520 nm) was monitored by a Horiba FluoroMax-4 spectrofluorometer (HORIBA, USA). Furthermore, activated platelets were pre-treated with 100 μg mL^-1^ of eptifibatide to inhibit α_IIb_β_3_ integrins 5 min prior to the addition of NBD-PE and Rhod-PE coated nanoerythrosomes.

**1.13** **Selective fibrin lysis in a fibrin clot model**

300 mg agar was dissolved in a buffer mixture (15 mL of 0.05 M Tris-HCl buffer at pH 7.2 and 5 mL of 0.025 M CaCl_2_ solution) at 95 °C. 50 mg fibrinogen was dissolved in 10 mL of Tris-HCl buffer (0.05 M, pH 7.2). The agar solution was mixed with the fibrinogen solution, and then 10 μL thrombin (4.0 U mL^-1^) was added under stirring for 1 min. The resulting mixture was spread carefully on a transparent plastic plate and homogeneous gels were obtained at 37 °C after 4 h. Sample wells were created in the plate and 5 μL of plasminogen solution (1 mg mL^-1^) was then added into each sample well, followed by 10 μL of sheep activated platelets (1.0 × 10^8^ mL^-1^) and 50 µL of PBS buffer, mBiNE, NE@tPA, mBiNE@tPA and free tPA (equivalent tPA dose of 1 mg mL^-1^) were added into respective sample wells and incubated at 37 °C for 24 h. The area of the lysed zone in each well was measured to evaluate the degree of fibrin clot lysis.

**1.14 Selective** **clot lysis in a Halo blood clot model**

A clotting mixture (5 mL of buffer containing 66 mM Tris-HCl, 130 mM NaCl and 45 mM CaCl_2_; 5μL of 1 μM thrombin, pH 7.4) was freshly prepared. In a 96-well microplate, 5 µL of fresh clotting mixture was placed on one side of the well bottom, then 15 µL of whole sheep blood was added on the opposite side of the well bottom. Clotting was initiated by mixing the two drops with a pipette tip in a circular motion to form a homogenous halo shape of blood around the edge of the well bottom, leaving the centre area empty. The microplate was covered and incubated at 37 ^o^C for 30 min. After clot formation, 80 μL mBiNE, NE@tPA, mBiNE@tPA and free tPA (equivalent tPA dose of 1 mg mL^-1^) were added simultaneously into respective wells containing halo clots. The dissolution of the halo clots was determined with a plate reader GloMax-Multi Microplate Multimode Reader (Promega, USA) by measuring the absorbance at 495 nm at 37 ^o^C. The clot degradation results in erythrocytes progressively covering the centre of the well over time. Halo thrombi treated with 80 μL of PBS at pH 7.4 were used as a negative control. 15 μL blood mixed with 85 μL of PBS (no halo clots) were used as a positive control. The percentage of clot dissolution was calculated according to the following equation:

$\%Clot lysis= \frac{As- A_{n}}{A_{p}- A_{n}}\times100$	(S5)

where *A_s_* is the absorbance of halo clots treated with samples; *A_n_* is the absorbance of the negative control; *A_P_* is the absorbance of the positive control.

**1.15 Pharmacokinetic and biodistribution studies**

SD rats were anesthetized by sodium pentobarbital and randomly divided into three groups (n = 3). The FITC-labeled tPA, NE@tPA and mBiNE@tPA solutions were intravenously injected at the equivalent tPA dose of 10 mg kg^-1^. After treatment, about three or four drops of blood (~0.3 mL) was drawn from the eye socket at 1, 3, 5, 8, 10, 15, 30 and 60 min for the free tPA group, while at 1, 15, 30, 60, 120, 240, 720 and 1440 min for the NE@tPA and mBiNE@tPA groups. Plasma was separated by centrifugation at 3500 × g for 10 min. 150 μL of plasma was taken for fluorescence measurements by a plate reader (BioTek Epoch, USA) at 520 nm. At 6 h post administration, the hearts, livers, spleens, lungs and kidneys were collected for the biodistribution studies. All tissues were rinsed in saline, dried carefully and homogenized. 150 μL of the tissue samples were taken for fluorescence measurements by a plate reader. The concentrations of tPA in the blood or tissue samples were calculated by using the standard curve. Half-lives (t_1/2_) were calculated according to the following equation:

$K_{e}= \frac{lnC_{1}-lnC_{2}}{(t_{2}-t_{1})}$	(S6)

$t_{1/2}= \frac{0.693}{K_{e}}$	(S7)

where K_e_ (h^-1^) is the elimination rate constant, and C_1_ (mg L^-1^) and C_2_ (mg L^-1^) are the tPA concentrations at time t_1_ and t_2_, respectively.

**1.16 Thrombus targeting and penetration**

The tails of Kunming (KM) mice containing thrombi were separated and stored in 4% paraformaldehyde solution at 4 ^o^C for 48 h. The tails were then decalcified and embedded in paraffin and cut into small slices. The resulting slices were incubated with the FITC-labeled or Cy5.5-labeled free tPA, NE@tPA, mBiNE@tPA, Lip@tPA, and cRGD-Lip@tPA (equivalent tPA dose of 25 μg mL^-1^), respectively, at 37 ^o^C for 35 min. The resulting slices were washed three times with PBS under shaking (5 min per time) before being mounted and imaged with an ECLIPSE 80i fluorescence microscope (Nikon, Japan).

**1.17 *In vivo* biosafety assessments**

After 7 days of treatment with PBS buffer, mBiNE, NE@tPA, mBiNE@tPA and free tPA (equivalent tPA dose of 10 mg kg^-1^), respectively, KM mice were sacrificed, the blood was collected, and the major organs (hearts, livers, spleens, lungs, and kidneys) were harvested. The hematology data was measured by a BC-30Vet automatic blood cell analyzer (Mindray, China). The serum biochemistry was assessed by a Chemray 240 automatic chemistry analyzer (Rayto, China). The major organs were embedded in paraffin and small slices were cut off for H&E staining for biosafety evaluation.

**1.18 Coagulation studies**

Thromboelastography (TEG) assay: Healthy SD rats were divided randomly into three groups (n = 3) and intravenously injected with PBS, NE@tPA and mBiNE@tPA (equivalent tPA dose of 10 mg kg^-1^), respectively. At 24 h post-injection, rats were anesthetized by intraperitoneal injection with sodium pentobarbital (50 mg kg^-1^). Whole blood was taken from the abdominal aorta of the rats and collected in 3.8 % (w/v) sodium citrate (volume ratio of blood to citrate at 1:9). 1 mL of citrated blood was transferred to a vial containing 20 μL kaolin (0.3 mg mL^-1^). Subsequently, 340 μL of the above kaolin-activated blood was moved into a 37 ^o^C pre-warmed TEG hemostasis system disposable plastic cup and pin (clear cup) containing 20 μL calcium chloride (0.2 M), and coagulation parameters of the formed whole blood clots were studied using the Haemoscope TEG 5000 Thrombelastograph Hemostasis Analyzer (Haemonetics Corp., USA) according to the manufacturer’s instructions. Major coagulation parameters including R time (time to formation of initial fibrin threads), K time (time until a thrombus reaches a strength of 20 mm), Alpha angle (rapidity with which a thrombus forms), and maximum amplitude (MA, maximum strength of a thrombus) were analyzed and compared among different groups.

Plasma coagulation: Whole blood was taken from the abdominal aorta of the SD rats (or KM mice) and collected in a tube containing 3.8 % (w/v) sodium citrate as an anticoagulant (volume ratio of blood to citrate at 1:9). The blood was centrifuged at 3,000 rpm (1,238 ×g) for 12 min at 4 ^o^C. The supernatant was collected to obtain plasma. Four plasma coagulation indicators, including activated partial thromboplastin time (APTT), prothrombin time (PT), thrombin time (TT), and fibrinogen (FBG), were examined and recorded by an RAC-1830 automatic coagulometer (Rayto Life and Analytical Sciences Co., Ltd, China). The rats (or mice) injected with PBS were used as control group.

**1.19 Tail bleeding assay**

Healthy KM mice were divided randomly into five groups (n = 5) and intravenously injected with PBS buffer, mBiNE, NE@tPA, mBiNE@tPA and free tPA (equivalent tPA dose of 10 mg kg^-1^), respectively. At 2 h post-injection, the mice were anesthetized and placed in prone position. The distal 1-cm section of the tail was cut off and then submerged into the pre-warmed saline solution at 37 ^o^C. Mice were observed for 25 min. The bleeding time was defined as the time for the transected tail to stop bleeding for at least 10 s. To calculate the bleeding volume, blood cells were centrifuged at 4000 × g for 10 min. Cell pellets were resuspended in 1 mL of 1% Triton X-100 and the bleeding volume was measured according to the hemoglobin concentrations compared to the standard curve.

**2. Supplementary** **computational modeling methods**

To simulate *in vivo* mouse tail thrombolysis scenarios, we adapted our previous model^[3]^ by incorporating the systematic PK-PD model into a 3D mouse tail thrombolysis model. Additionally, the flow and species transport mechanisms have been adjusted to accurately depict blood flow and circulation within the mouse tail.

**2.1 Reaction kinetics**

The model incorporates three categories of biochemical reactions. The one-compartment systemic PK-PD model includes plasma reactions and tPA release reactions. In the mouse tail thrombolysis model, all three categories of reactions are represented. Supplementary Table S1 lists the kinetic parameters for all reactions.

**2.1.1 Plasma reactions**

$\text{tPA}+\text{PLG}\underset{\to}{K_{M,PLG}\text{ \& }k_{cat,PLG}}\text{tPA}+\text{PLS},r_{tPA-PLG}=\frac{k_{cat,PLG}C_{tPA}C_{PLG}}{K_{M,PLG}+C_{PLG}}$	(S8)

$$\text{PLS}+\text{AP}\underset{\rightleftarrows}{k_{AP,f}k_{AP,r}}\text{PLS}-\text{AP}\underset{\to}{k_{cat,AP}}\text{inactive, }r_{AP,1}=k_{AP,f}C_{PLS}C_{AP}-k_{AP,r}C_{PLS-AP}$$

$r_{AP,2}=k_{cat,AP}C_{PLS-AP}$	(S9)

$\text{PLS}+\text{FBG}\underset{\to}{K_{M,FBG}\text{ \& }k_{cat,FBG}}\text{PLS}+\text{FDP},r_{PLS-FBG}=\frac{k_{cat,FBG}C_{PLS}C_{FBG}}{K_{M,FBG}+C_{FBG}}$	(S10)

$\text{PLS}+\text{MG}\underset{\to}{k_{MG}}\text{inactive},r_{MG}=k_{MG}C_{PLS}C_{MG}$	(S11)

$\text{tPA}+\text{PAI}\underset{\to}{k_{PAI}}\text{inactive},r_{PAI}=k_{PAI}C_{tPA}C_{PAI}$	(S12)

**2.1.2 Fibrinolytic reactions in the clot phase**

$\text{tPA}+F\underset{\rightleftarrows}{k_{a,tPA}k_{d,tPA}}\text{tPA-F},r_{tPA-F}=k_{a,tPA}C_{tPA}n_{free}-k_{d,tPA}n_{tPA}$	(S13)

$\text{PLG}+F\underset{\rightleftarrows}{k_{a,PLG}k_{d,PLG}}\text{PLG-F},r_{PLG-F}=k_{a,PLG}C_{PLG}n_{free}-k_{d,PLG}n_{PLG}$	(S14)

$\text{PLS}+F\underset{\rightleftarrows}{k_{a,PLS}k_{d,PLS}}\text{PLS-F},r_{PLS-F}=k_{a,PLS}C_{PLS}n_{free}-k_{d,PLS}n_{PLS}$	(S15)

$\text{tPA-F}+\text{PLG-F}\underset{\to}{K_{M}\text{ \& }k_{M,cat}}\text{tPA-F}+\text{PLS-F},r_{PLS,MM}=\frac{k_{cat}n_{PLG}n_{tPA}}{K_{M}\left( 1-\varepsilon_{clot} \right)+n_{PLG}}$	(S16)

$\text{PLS-F}\underset{\to}{k_{deg}}\text{PLS-}\text{F}_{\text{lysed}},r_{deg}=k_{deg}\gamma n_{PLS}$	(S17)

$\text{PLS-}\text{F}_{\text{lysed}}\underset{\to}{k_{d,PLS}}\text{PLS}+F_{\text{lysed}},r_{PLS,return}=k_{d,PLS}n_{deg}$	(S18)

**2.1.3** **Triggered tPA release reactions**

$\text{m}\text{B}\text{i}\text{NE}\text{@tPA}+\text{INT}\underset{\rightleftarrows}{k_{a\text{ }}k_{d}}\text{INT-}\text{m}\text{B}\text{i}\text{NE,}\text{ }r_{mBiNE@tPA-INT}=k_{a,\text{m}\text{B}\text{i}\text{N}\text{E}}C_{\text{m}\text{B}\text{i}\text{N}\text{E}}C_{INT,free}-k_{d,\text{m}\text{B}\text{i}\text{NE}}C_{INT-\text{m}\text{B}\text{i}\text{NE}\text{@tPA}}$	(S19)

$\text{INT-}\text{m}\text{B}\text{i}\text{NE}\text{@tPA}\underset{\to}{k_{rel}}\nu_{rel}\text{tPA}+\text{INT-}\text{mBiNE}\text{,}\text{ } r_{rel}=k_{rel}C_{\text{m}\text{B}\text{i}\text{NE}\text{@tPA}-INT}$	(S20)

$\text{mBiNE}+\text{INT}\underset{\rightleftarrows}{k_{a,}k_{d,}}\text{INT-}\text{mBiNE}$, $r_{\text{m}\text{B}\text{i}\text{NE}-INT}=k_{a,\text{m}\text{B}\text{i}\text{N}\text{E}}C_{\text{m}\text{B}\text{i}\text{N}\text{E}}C_{INT,free}-k_{d,\text{m}\text{B}\text{i}\text{NE}}C_{INT-mBiNE}$	(S21)

$\text{m}\text{B}\text{i}\text{NE}\text{@tPA}\underset{\leftrightarrow}{K_{leak}}\nu_{rel}\text{tPA}+\text{mBiNE}, r_{leak}=K_{leak}\left( \nu_{rel}C_{\text{m}\text{B}\text{i}\text{NE}\text{@tPA}}-C_{tPA} \right)$	(S22)

**Table S1.** Reaction kinetic parameters.

| **Symbol** | **Description** | **Value** | **Unit** | **Source** |
| --- | --- | --- | --- | --- |
| *k_a,tPA_* | Adsorption coefficient for tPA | 0.01 | μM^-1^ s^-1^ | [4] |
| *k_d,tPA_* | Desorption coefficient for tPA | 0.0058 | s^-1^ | [4] |
| *k_a,PLG_* | Adsorption coefficient for PLG | 0.1 | μM^-1^ s^-1^ | [5] |
| *k_d,PLG_* | Desorption coefficient for PLG | 3.8 | s^-1^ | [6] |
| *k_a,PLS_* | Adsorption coefficient for PLS | 0.1 | μM^-1^ s^-1^ | [5] |
| *k_d,PLS_* | Desorption coefficient for PLS | 0.05 | s^-1^ | [5] |
| *K_M_* | Michaelis constant for PLG conversion in the bound phase | 0.1 | μM | [7] |
| *k_cat_* | Michaelis reaction rate coefficient for PLG conversion in the bound phase | 0.3 | s^-1^ | [8] |
| *k_deg_* | Lysis coefficient | 2.178 | s^-1^ | [8] |
| *1/γ* | Cuts needed for PLS to cut 1 fibrin unit | 10 | - | [5] |
| *k_a_* | Adsorption coefficient for mBiNE$\text{@tPA}$ | 1.38×10^-2^ | μM^-1^ s^-1^ | Our experiment |
| *k_d_* | Desorption coefficient for $\text{m}\text{B}\text{i}\text{NE}\text{@tPA}$ | 7.59×10^-4^ | s^-1^ | Our experiment |
| *k_rel_* | Triggered release rate constant | 0.205 | s^-1^ | Our experiment |
| *K_leak_* | Leakage rate constant | 5.93×10^-9^ | s^-1^ | Our experiment |
| *K_M,PLG_* | Michaelis constant for PLG conversion in the free phase | 28.03 | μM | [9] |
| *k_cat,PLG_* | Michaelis reaction rate coefficient for PLG conversion in the free phase | 0.3 | s^-1^ | [9] |
| *K_M,FBG_* | Michaelis constant for FBG conversion in the free phase | 55 | μM | [9] |
| *k_cat,FBG_* | Michaelis reaction rate coefficient for FBG conversion in the free phase | 250 | s^-1^ | [9] |
| *k_AP,f_* | Forward reaction constant for AP and PLS | 10 | μM^-1^ s^-1^ | [9] |
| *k_AP,r_* | Reverse reaction constant for AP and PLS | 0.0021 | s^-1^ | [9] |
| *k_cat,AP_* | Inactivation rate constant by AP | 0.004 | s^-1^ | [9] |
| *k_MG_* | Reaction constant for MG and PLS | 0.35 | μM^-1^ s^-1^ | [10] |
| *k_PAI_* | Reaction constant for tPA and PAI | 37 | μM^-1^ s^-1^ | [11] |
| *ν_rel_* | Stochiometric coefficient for tPA and mBiNE@tPA reaction | 3394 | - | Our experiment |

**2.****2 Clot properties in computational modeling**

**2.2.1** **geometry of mouse tail thrombus**

The dimension of the mouse tail thrombus tip end, the dimension of the mouse tail thrombus base, and the cross-section of the mouse tail thrombus in ZY direction are shown in Supplementary Figure S23.

**2.2.2** **Initial clot properties and time-varying clot properties**

The initial clot volume fraction, clot permeability, acitivated platelet concentration, and fibrin binding site concentration are calculated based on the initial volume fractions of fibrin fibre network (FBR) *Φf,0* and activated platelets (PLT) *Φp,0* in the clot. The detail about the fibrin fibre network and methods for calculating initial and time-varying clot properties such as the extent of lysis ($E_{L})$ can be found in our group’s previous work. ^[3]^

**2.****3 Mouse tail model equations for flow and species transport in computational modeling**

The species transport is governed by the convection-reaction-diffusion equation. Since the pressure in the occluded mousetail is unknown, A constant velocity of 0.11 m s^-1^ is given, which is the blood velocity of the caudal artery from the literature. ^[12]^ According to the blood flow intensity measurement, we assumed that there is no convection flow in the thrombus:

$\frac{\partial C}{\partial t}=D_{j}\nabla^{2}C_{j}-S\cdot\nabla\mathbf{u}C_{j}+r_{total,j},$ $r_{total,j}=R_{j}^{clot}+R_{j}^{plasma}$	(S23)

where $j=mBiNE@tPA, mBiNE,PLT, tPA,PLG, PLS,FBG, AP,PLS-AP,FBG,MG and PAI$,

S = 1 in clot dissolved region ($E_{L}>E_{L,crit})$,

S = 0 in clot undissolved region ($E_{L}<E_{L,crit})$,

where $\varepsilon$ is the porosity of the clot, $R_{j}^{clot}$is the clot phase reaction rate, $R_{j}^{plasma}$ is the plasma phase reaction rate, $R_{j}^{total}$is the total reaction rate, $D_{j}$ is the diffusivity of fibrinolysis proteins and mBiNE@tPA in the clot, and **u** is the flow velocity in the clot. Since there is no physical flow outlet in the mousetail model, we assumed that the collateral blood circulation would be restored at the clot dissolved clot region, and therefore, all species can flow out of the mousetail model at the interface between the dissolved clot and undissolved clot region. All bound and free phase species at the front face of the thrombus will be purged once the region is dissolved completely$(E_{L}>E_{L,crit})$.

**2.4 Additional in vitro model simulation details**

For the in vitro experiment simulations, the initial species concentrations are detailed in Supplementary Table S2. A system of ordinary differential equations was numerically solved using the ode23s solver in MATLAB 2019b. The simulations employed a time step of 1 second.

**Table S2.** Initial condition for static model.

| **Description** | **Selected value** | **Typical value in the plasma [Source]** |
| --- | --- | --- |
| Initial volume fraction of fibrin fiber, *Φf,0* | 0.03 | Blood clot overall porosity 0.9-0.99 [3] |
| Initial volume fraction of platelets, *Φp,0* | 0.01 | Blood clot overall porosity 0.9-0.99 [3] |
| Initial concentration of tPA  (or mBiNE@tPA) | 2.152 μM | Our experiment |
| Initial concentration of PLG | 2.2 μM | 2.2 μM [3] |
| Initial concentration of PLS | 0 μM | 0 μM [3] |
| Initial concentration of FBG | 8 μM | 8 μM [3] |
| Initial concentration of AP  Initial concentration of MG | 0.5 μM  1.5 μM | 0.5 μM [3]  1.5 μM [3] |
| Initial concentration of PAI | 5.23×10^-4^ μM | 5.23e-04 μM [13] |

**2.5 Calculation of systemic PK-PD model parameters**

Elimination constant: $k_{el,i}=\frac{\ln2}{t_{1/2,i}}$ (S24)

Generation rate:(S25)

The half-life time t_1/2_ of mBiNE@tPA and free tPA is estimated by fitting the systemic PK-PD model to the experimental measurement of the plasma level in the SD rat. Comparison between the best-fitting prediction and experiment data is shown in Supplementary Figs. 16a and 16b.

The distribution volume of tPA and mBiNE@tPA is calculated as: $V_{tPA/mBiNE@tPA}=\frac{Dose adminstered}{{C_{mBiNE@tPA /tPA}}_{t=0}}$, where ${C_{mBiNE@tPA/tPA}}_{t=0}$ is estimated by extrapolation to time = 0 on a plasma concentration vs. time curve. Reaction kinetic parameters are listed in Supplementary Table S1. Other parameters applied to the systemic PK-PD model are shown in Supplementary Table S3.

**2.6** **Additional local thrombolysis model simulation detail**

The computational mesh used for the concentric cone geometry thrombus, as depicted in Supplementary Figure S24, was created with Ansys Design Modeler. This structured mesh is composed of 13,620 hexahedral elements and is adequately refined to guarantee a mesh-independent solution. The boundary and initial conditions, along with the model parameters, are presented in Supplementary Tables S3 and S4, respectively. The model equations were adapted using the ScalarTransportFoam solver from OpenFOAM 9.0, a widely used open-source CFD software. A time step of 0.05 seconds was implemented. For the simulation of 7 days thrombolysis, results were recorded every 6 h, and for the 1-hour thrombolysis simulation, data was saved every minute.

**Table S3.** Modeling parameters applied in the systemic PK-PD model and local thrombolysis model.

| **Symbol** | **Description** | **Value** | **Unit** | **Source** |
| --- | --- | --- | --- | --- |
| *C_AP,0_* | Initial AP concentration | 1 | µM | [14] |
| *C_FBG,0_* | Initial FBG concentration | 7 | µM | [15] |
| *C_PLG,0_* | Initial PLG concentration | 0.94 | µM | [16] |
| *C_PLS,0_* | Initial PLS concentration | 0 | µM | [3] |
| *C_tPA,0_* | Initial tPA concentration | 0.05×10^-3^ | µM | [3] |
| *C_MG,0_* | Initial MG concentration | 0.04 | μM | [17] |
| *C_PAI,0_* | Initial PAI concentration | 1.11×10^−4^ | μM | [18] |
| *C_PLT,0_* | Initial PLT concentration | 9×10^11^ | L^-1^ | [19] |
| *t_1/2,AP_* | Half-life time of AP | 2.64 | day | [20] |
| *t_1/2,FBG_* | Half-life time of FBG | 4.14 | day | [21] |
| *t_1/2,PLG_* | Half-life time of PLG | 2.2 | day | [22] |
| *t_1/2,PLS_* | Half-life time of PLS | 0.1 | s | [23] |
| *t_1/2,tPA_* | Half-life time of tPA | 2.32 | min | our experiment |
| *t_1/2,PAI_* | Half-life time of PAI | 1.5 | hr | [24] |
| *t_1/2,MG_* | Half-life time of MG | 3 | min | [25] |
| *t_1/2,mBiNE@tPA_* | Half-life time of mBiNE@tPA | 110 | min | our experiment |
| *V_mBiNE@tPA_* | Plasma distribution volume of mBiNE@tPA | 1.4×10^-3^ | L | our experiment |
| *V_tPA_* | Plasma distribution volume of tPA | 2.2×10^-3^ | L | our experiment |
| *M_w,tPA_* | tPA molecular weight | 59.04 | mg µmol^-1^ | - |
| *M_w,mBiNE@tPA_* | mBiNE@tPA molecular weight | *v_rel_*×*M_w,tPA_* | mg µmol^-1^ | - |
| *D_proteins_* | Diffusivity of plasma proteins | 5×10^-11^ | m^2^ s^-1^ | [26] |
| *D_mBiNE@tPA_* | Diffusivity of mBiNE@tPA | 4.11×10^-12^ | m^2^ s^-1^ | [27] |
| *D_PLT_* | Diffusivity of PLT | 3.1×10^-14^ | m^2^ s^-1^ | [28] |
| *E_L,cirt_* | Critical extent of lysis | 0.95 | - | [29] |
| *N_INT_* | Number of integrins expressed upon activation of platelets | 45000 | numbers | [30] |
| *P_max_* | Max. number of platelets per unit volume | 1.2×10^14^ | numbers | [31] |

**Table S4.** Boundary condition.

| location | Velocity | Concentration |
| --- | --- | --- |
| Inlet | 0.11 m s^-1^ | Time-varying concentration $C_{i,}(t=all,x=0)$ from the systemic PK-PD model |
| Wall | No slip | Zero gradient |

**3. Supplementary figures**

**Figure S1.** (a) Schematic of preparation of erythrocyte ghosts through hypotonic treatment with 0.2 mM EDTA solution for a total of seven cycles. (b) Sodium dodecyl sulfate-polyacrylamide gel electrophoresis (SDS-PAGE) was used to identify membrane proteins of erythrocyte ghosts by comparing to a pre-stained protein ladder. (c) A table of erythrocyte membrane proteins reproduced from reference ^[32]^ with permission under an open access Creative Common CC BY license from MPDI publisher. In the Figure S1b, thick bands that represent spectrin (α and β chains) were seen between 260 and 160 kDa, demonstrating that not only were the proteins present but that there were high concentrations of both in the sample. A thinner band, which was seen under the two bands representing the α and β chains of spectrin, has a molecular weight similar to ankylin. The band was not as intense as the other two bands indicating that the protein was present but at a lower concentration compared to the other two spectrin bands. There were several bands present between 110 and 80 kDa. These bands had similar molecular weights as band 3 (AE1) and band 4.1 proteins, with the thicker one associated with band 4.1. The protein band 4.2 had a molecular weight that was similar to the two thicker bands between 80 and 60 kDa of the ladder. The darker band between 50 and 40 kDa had a molecular weight consistent with actin. These suggested that all proteins associated with the erythrocyte membrane, as listed in Figure S1c, were found in the sample of erythrocyte ghosts.

**Figure S2.** Western blotting analysis of membrane-specific protein markers, CD47 and CD235a, in the erythrocyte membrane or mBiNE@tPA (n=3).

**Figure S3.** Full-size western blotting images for Figure S2, showing (a) CD47, (b) CD235a, and (c) *β*-actin in the erythrocyte membrane or mBiNE@tPA (n=3).

**Figure S4.** (a) Mean hydrodynamic diameters and (b) polydispersity index (PDI) of NE, mBiNE, NE@tPA and mBiNE@tPA in pH 7.4 PBS buffer at 25 ^o^C. Data are presented as the average ± SD (n=3). Statistical analysis was performed by the ANOVA test (ns, not significant).

**Figure S5.** (a) Encapsulation efficiencies and (b) loading capacities of NE@tPA and mBiNE@tPA. Data are presented as the average ± SD (n=3). Statistical analysis was performed by the Student’s t-test (ns, not significant).

**Figure S6.** Zeta potentials of the NE@tPA and mBiNE@tPA in the presence of 50% (v/v) FBS solution in PBS 7.4 buffer during 24 h of incubation in water bath at 37 ^o^C under shaking at 120 rpm. Data are presented as the average ± SD (n=3).

**Figure S7.** Schematic of a benchtop pressure-driven extruder for scalable production of mBiNE@tPA. This nitrogen-driven extruder with a working volume of one to ten milliliters of sample was designed with the help of the Mr. Anthony Meredith at the Mechanical Workshop in the Chemical Engineering Department, Imperial College London.

**Figure S8.** Residual tPA activity after 30 min of trypsin treatment, as measured by the S‑2251 chromogenic substrate assay. Data are presented as the average ± SD (n = 3). Statistical analysis was performed by the ANOVA test (ns, not significant).

**Figure S9.** Flow cytometry histogram profiles of resting platelets incubated with the FITC-labeled tPA, FITC-labeled NE@tPA and mBiNE@tPA (equivalent tPA concentration of 0.2 mg mL^-1^) at 37 ^o^C for 4 h, respectively. Treatment with PBS buffer at pH 7.4 under the same conditions was used as control.

**Figure S10.** Flow cytometry histogram profiles of activated platelets incubated with the FITC-labeled tPA, FITC-labeled NE@tPA and mBiNE@tPA (equivalent tPA concentration of 0.2 mg mL^-1^) at 37 ^o^C for 4 h, respectively. Treatment with PBS buffer at pH 7.4 under the same conditions was used as control.

**Figure S11.** Flow cytometric mean fluorescence intensities (MFI) of resting or activated platelets treated with FITC-labeled tPA (tPA dose of 0.2 mg mL^-1^) at 37 ^o^C for 4 h, respectively, relative to control treated with pH 7.4 PBS. Data are presented as the average ± SD (n = 3). Statistical analysis was performed by the Student’s t-test (ns, not significant).

**Figure S12.** Relative fluorescence intensities of activated platelets incubated with the FITC-labeled mBiNE@tPA containing different cRGD densities at 37 ^o^C for 4 h, as measured by flow cytometry. Data are presented as the average ± SD (n=3).

**Figure S13.** tPA release profile of mBiNE@tPA after incubation with different concentrations of activated platelets for 6 h. Data are presented as the average ± SD (n=3). Statistical analysis was performed by the ANOVA test (*****p* < 0.0001 and ns, not significant).

**Figure S14.** (a) Particle concentration of mBiNE@tPA (equivalent tPA concentration of 0.53 mg mL^-1^) in pH 7.4 PBS buffer, as measured by nanoparticle tracking analysis (NTA). In NTA, 1 mL of the mBiNE@tPA dispersion contained 1.19 × 10^12^ particles (i.e. 1.98 × 10^-12^ mol) and 6.72 × 10^-9^ mol of tPA. Thus, the average molar ratio between tPA and nanoerythrosome was 3,394. (b) cRGD lipid concentration in mBiNE@tPA. (c) cRGD surface density on mBiNE@tPA. The calculated cRGD lipid concentration was 7.86 × 10^-8^ mol mL^-1^. Therefore, the calculated cRGD surface density on mBiNE@tPA was 3.96 × 10^4^ molecules per particle. Data are presented as the average ± SD (n = 3).

**Figure S15.** Representative bright-field image of the fibrillar collagen-coated channel. Scale bar, 100 μm.

**Figure S16.** (a) Mean hydrodynamic diameters and (b) zeta potential of Lip@tPA and cRGD-Lip@tPA in pH 7.4 PBS buffer at 25 ^o^C. Fluorescence images and intensity profiles of the KM mouse tail thrombus slices, which were collected and stained with (c) FITC-labeled Lip@tPA or (d) cRGD-Lip@tPA at an equivalent tPA dose of 2.5 μg mL^-1^, respectively, at 37 °C for 35 min. Dotted lines indicate the cross-sections used to produce the intensity profiles with the use of Fiji. Data are presented as the average ± SD (n=3).

**Figure S17.** (a) Fluorescence images of KM mouse tail-thrombus slices which were collected and stained with Cy5.5-labeled free tPA, Lip@tPA, cRGD-Lip@tPA, NE@tPA and mBiNE@tPA (equivalent tPA dose of 25 μg mL^-1^), respectively, at 37 °C for 35 min. Scale bar, 100 μm. (b) MFI in the fluorescence images of S17a, as analyzed by Fiji. Data are presented as the average ± SD (n = 3). Statistical analysis was performed by ANOVA test (*p < 0.05, **p < 0.01, ***p < 0.001, and ****p < 0.0001).

**Figure S18.** Initial lengths of black tail thrombi in seven randomly divided mouse groups before intravenous injection with PBS buffer, mBiNE, NE@tPA, mBiNE@tPA, free tPA, Lip@tPA, and cRGD-Lip@tPA, respectively. Data are presented as the average ± SD (n = 5). Statistical analysis was performed by ANOVA test (ns, not significant).

**Figure S19.** (a) Illustration of the ROI_4-cm_, a 4-cm location of the mouse tail focused by a laser doppler flowmeter to evaluate the blood flow. (b) Mean blood flow intensities at the ROI_4-cm_ in seven randomly divided mouse groups before intravenous injection with PBS buffer, mBiNE, NE@tPA, mBiNE@tPA, free tPA, Lip@tPA, and cRGD-Lip@tPA, respectively. Data are presented as the average ± SD (n = 5). Statistical analysis was performed by ANOVA test (ns, not significant).

**Figure S20.** Black tail thrombus length losses in tail thrombus-bearing KM mice after 1, 6, 12, 18, and 24 h of treatment. Data are presented as the average ± SD (n = 5). Statistical analysis was performed by the ANOVA test (****p < 0.0001).

**Figure S21.** Flow chart of the solution procedure for the systemic PK-PD model and local thrombolysis model. Input information required for solving each model is specified in the black boxes.

**Figure S22.** Comparison of model predictions and experimental results of (a) free tPA and (b) mBiNE@tPA concentrations in plasma.

 **Figure S23.** The dimension of (a) mouse tail thrombus tip end and (b) mouse tail thrombus base. (c) The cross section of mouse tail thrombus in ZY direction.

**Figure S24.** Geometry of the local mouse tail thrombus model.

**Figure S25.** Plasma concentration profiles of fibrinolysis species after mBiNE@tPA treatment in the mouse tail thrombosis model.

**Figure S26.** Plasma concentration profiles of fibrinolysis species after free tPA treatment in the mouse tail thrombosis model.

**Figure S2****7.** Concentration variations of (a) plasma phase tPA (C_tPA_), (b) fibrin-bound tPA (n_tPA_), and (c) fibrin-bound PLS (n_PLS_) during 7 days of treatment with mBiNE@tPA and free tPA, respectively, in the mouse tail thrombosis model with a fibrin volume fraction at *Φ_f_* = 0.0015 and a platelet volume fraction *Φ_p_* = 0.05. Note that the data are sampled every 6 h, which means that concentration variations within 6 h may be omitted.

**Figure S28.** Comparison of the mBiNE@tPA induced thrombolysis in platelet-rich (*Φ_p_* = 0.05) and platelet-poor (*Φ_p_* = 0.01) clot at *Φ_f_* = 0.01 in the mouse tail thrombosis model.

**Figure S29.** Body weights of KM mice after 7 days of treatment with PBS buffer, mBiNE, NE@tPA, mBiNE@tPA, free tPA, Lip@tPA, and cRGD-Lip@tPA (equivalent tPA dose of 10 mg kg^-1^), respectively. Data are presented as the average ± SD (n = 5).

**Figure S30.** Representative H&E staining photomicrographs of major organs (heart, liver, spleen, lung, and kidney) in KM mice after 7 days of treatment with PBS buffer, mBiNE, NE@tPA, mBiNE@tPA, free tPA, Lip@tPA, and cRGD-Lip@tPA (equivalent tPA dose of 10 mg kg^-1^), respectively. Scale bar, 50 μm.

**Figure S31.** Plasma coagulation indicators including APTT, activated partial thromboplastin time, PT, prothrombin time, TT, thrombin time, and FBG, fibrinogen. Intravenous injection of KM mice with PBS, NE@tPA and mBiNE@tPA (equivalent tPA dose of 10 mg kg^-1^), respectively, followed by measurements by coagulometry. Data are presented as the average ± SD (n = 5).

**Figure S32.** (a) Bleeding time, (b) bleeding volume, and (c) bleeding index of the tail-transected KM mice at 2 h post intravenous injection with PBS buffer, mBiNE, NE@tPA, mBiNE@tPA, and free tPA (equivalent tPA dose of 0.9 mg kg^-1^), respectively. The bleeding index was defined by the product of bleeding time (min) and bleeding volume (mL). Data are presented as the average ± SD (n =5). Statistical analysis was performed by the ANOVA test (****p < 0.0001 and ns, not significant).

**4. Supplementary movies**

**Movie S1.** Temporal and spatial variations of the extent of thrombolysis during 7 days of free tPA treatment in the mouse tail thrombosis model with *Φ_f_* = 0.0015 and *Φ_p_* = 0.05.

**Movie S2.** Temporal and spatial variations of the extent of thrombolysis during 7 days of mBiNE@tPA treatment in the mouse tail thrombosis model with *Φ_f_* = 0.0015 and *Φ_p_* = 0.05.

**5. References**

[1] H. Katas, Z. Hussain, S. A. Awang, Bovine serum albumin-loaded chitosan/dextran nanoparticles: preparation and evaluation of ex vivo colloidal stability in serum, J. Nanomater. 2013 (2013) 1−9.
[2] Y. Huang, J. Wang, Y. Guo, S. Y. Park, H. Yang, A. Lu, Y. Li, R. Chen, Selective binding of cationic fibrinogen-mimicking chitosan nanoparticles to activated platelets and efficient drug release for antithrombotic therapy, Int. J. Biol. Macromol. 268 (2024) 131742.
[3] B. Gu, Y. Huang, E. L. Manchester, A. D. Hughes, S. A. M. Thom, R. Chen, X. Y. Xu, Multiphysics modelling and simulation of thrombolysis via activated platelet-targeted nanomedicine, Pharm. Res. 39 (2022) 41−56.
[4] S. S. Husain, A. A. Hasan, A. Z. Budzynski, Differences between binding of one-chain and two-chain tissue plasminogen activators to non-cross-linked and cross-linked fibrin clots, Blood 74 (1989) 999−1006.
[5] D. M. Wootton, A. S. Popel, B. Rita Alevriadou, An experimental and theoretical study on the dissolution of mural fibrin clots by tissue-type plasminogen activator, Biotechnol. Bioeng. 77 (2002) 405−419.
[6] M. A. Lucas, L. J. Fretto, P. A. McKee, The binding of human plasminogen to fibrin and fibrinogen, J. Biol. Chem. 258 (1983) 4249−4256.
[7] M. Hoylaerts, D. C. Rijken, H. R. Lijnen, D. Collen, Kinetics of the activation of plasminogen by human tissue plasminogen activator. role of fibrin, J. Biol. Chem. 257 (1982) 2912−2919.
[8] S. Kunitada, G. A. FitzGerald, D. J. Fitzgerald, Inhibition of clot lysis and decreased binding of tissue-type plasminogen activator as a consequence of clot retraction, Blood 79 (1992) 1420−1427.
[9] B. E. Sobel, R. W. Gross, A. K. Robinson, Thrombolysis, clot selectivity, and kinetics, Circulation 70 (1984) 160−164.
[10] A. J. Tiefenbrunn, R. A. Graor, A. K. Robison, F. V. Lucas, A. Hotchkiss, B. E. Sobel, Pharmacodynamics of tissue-type plasminogen activator characterized by computer-assisted simulation, Circulation 73 (1986) 1291−1299.
[11] W. L. Chandler, M. C. Alessi, M. F. Aillaud, P. Henderson, V. Pague, I. Juhan-Vague, Clearance of tissue plasminogen activator (tpa) and tpa/plasminogen activator inhibitor type 1 (pai-1) complex: relationship to elevated tpa antigen in patients with high pai-1 activity levels, Circulation 96 (1997) 761−768.
[12] X. Tan, R. Patil, P. Bartosik, J. M. Runnels, C. P. Lin, M. Niedre, In vivo flow cytometry of extremely rare circulating cells, Sci. Rep. 9 (2019) 1−11.
[13] D. E. Vaughan, Pai-1 and atherothrombosis, J. Thromb. Haemost. 3 (2005) 1879−1883.
[14] S. Singh, A. K. Houng, G. L. Reed, Venous stasis-induced fibrinolysis prevents thrombosis in mice: role of α2-antiplasmin, Blood 134 (2019) 970.
[15] K. R. Machlus, J. C. Cardenas, F. C. Church, A. S. Wolberg, Causal relationship between hyperfibrinogenemia, thrombosis, and resistance to thrombolysis in mice, Blood 117 (2011) 4953.
[16] S. Singh, A. K. Houng, D. Wang, G. L. Reed, Physiologic variations in blood plasminogen levels affect outcomes after acute cerebral thromboembolism in mice: a pathophysiologic role for microvascular thrombosis, J. Thromb. Haemost. 14 (2016) 1822.
[17] T. Jinbo, M. Motoki, S. Yamamoto, Comparative medicine variation of serum α 2-macroglobulin concentration in healthy rats and rats inoculated with staphylococcus aureus or subjected to surgery, Comparative Med. 51 (2001) 332−335.
[18] F. Samad, D. J. Loskutoff, Tissue distribution and regulation of plasminogen activator inhibitor-1 in obese mice, Mol. Med. 2 (1996) 568−582.
[19] T. H. Bugge, K. W. Kombrinck, M. J. Flick, C. C. Daugherty, M. J. Danton, J. L. Degen, Loss of fibrinogen rescues mice from the pleiotropic effects of plasminogen deficiency, Cell 87 (1996) 709−719.
[20] D. Collen, B. Wiman, Turnover of antiplasmin, the fast-acting plasmin inhibitor of plasma, Blood 53 (1979) 313−324.
[21] D. Collen, G. Tytgat, H. Claeys, R. Piessens, Metabolism and distribution of fibrinogen. i. fibrinogen turnover in physiological conditions in humans, Br. J. Haematol. 22 (1972) 681−700.
[22] D. Collen, G. Tytgat, H. Claeys, M. Verstraete, P. Wallén, Metabolism of plasminogen in healthy subjects: effect of tranexamic acid, J. Clin. Investig. 51 (1972) 1310−1318.
[23] R. Lijnen, D. Collen, Protease inhibitors of human plasma. α-2-antiplasmin, J. Med. 16 (1985) 225−284.
[24] N. V. Gorlatova, J. M. Cale, H. Elokdah, D. Li, K. Fan, M. Warnock, D. L. Crandall, D. A. Lawrence, Mechanism of inactivation of plasminogen activator inhibitor-1 by a small molecule inhibitor, J. Biol. Chem. 282 (2007) 9288−9296.
[25] P. E. H. Jensen, S. Humle Jørgensen, P. Datta, P. S. Sørensen, Significantly increased fractions of transformed to total α2-macroglobulin concentrations in plasma from patients with multiple sclerosis, Biochim. Biophys. Acta-Mol. Basis Dis. 1690 (2004) 203−207.
[26] G. D. O. Lowe, F. G. R. Fowkes, J. Dawes, P. T. Donnan, E. Housley, Blood viscosity, fibrinogen, and activation of coagulation and leukocytes in peripheral arterial disease and the normal population in the edinburgh artery study, Circulation 87 (1993) 1915−1920.
[27] H. Zhou, Z. Fan, P. K. Lemons, H. Cheng, A facile approach to functionalize cell membrane-coated nanoparticles, Theranostics 6 (2016) 1012.
[28] D. L. Bark, D. N. Ku, Platelet transport rates and binding kinetics at high shear over a thrombus, Biophys J. 105 (2013) 502−511.
[29] A. Piebalgs, B. Gu, D. Roi, K. Lobotesis, S. Thom, X. Y. Xu, Computational simulations of thrombolytic therapy in acute ischaemic stroke, Sci. Rep. 8 (2018) 1−13.
[30] B. D. Adair, C. O. Field, J. L. Alonso, J. P. Xiong, S. X. Deng, H. S. Ahn, E. Mashin, C. B. Clish, J. van Agthoven, M. Yeager, Y. Guo, D. A. Tess, D. W. Landry, M. Poncz, M. A. Arnaout, Platelet integrin αiibβ3 plays a key role in a venous thrombogenesis mouse model, Nat. Commun. 15 (2024) 8612.
[31] T. J. Stalker, E. A. Traxler, J. Wu, K. M. Wannemacher, S. L. Cermignano, R. Voronov, S. L. Diamond, L. F. Brass, Hierarchical organization in the hemostatic response and its relationship to the platelet-signaling network, Blood 121 (2013) 1875−1885.
[32] T. Aoki, A comprehensive review of our current understanding of red blood cell (rbc) glycoproteins, Membranes 7 (2017) 56.
